# Supplementary material for: Targeting SUMOylation promotes cBAF complex stabilization and disruption of the SS18::SSX transcriptome in synovial sarcoma
Source: Nat Commun. 2025 Nov 5;16:9761. doi: 10.1038/s41467-025-64665-8 (PMC12589557; doi:10.1038/s41467-025-64665-8)
Supplement: Supplementary file 1 — Supplementary Information [file 41467_2025_64665_MOESM1_ESM.pdf]

Supplementary information for:

Targeting SUMOylation promotes cBAF complex stabilization and disruption of the SS18::SSX transcriptome in Synovial Sarcoma

Konstantinos V. Floros[1,2], Carter K. Fairchild, Jr.[1,3,#], Jinxiu Li[4,#], Kun Zhang[1,2], Jane L. Roberts[1,2], Richard Kurupi[1,5], Durga Paudel[1,2], Yanli Xing[1,2], Bin Hu[6], Vita Kraskauskiene[6], Nayyerehalsadat Hosseini[6], Shanwei Shen[6], Melissa M. Inge[7,8], Kyllie Smith-Fry[4], Li Li[4], Afroditi Sotiriou[9,10,11], Krista M. Dalton[1,2], Asha Jose[1,12], Elsamani I. Abdelfadiel[1,2], Ronald D. Hill[1,2], Jamie M. Slaughter[1,2], Mayuri Shende[6], Madelyn R Lorenz[6], Noritaka Tanaka[13], Taisuke Kajino[13], Mary L. Nelson[4], Mandy R. Hinojosa[14,15], Victor A. Kehinde[1,2], Benjamin R. Belvin[1], Febri G. Sugiokto[16,17,18], Zhao Lai[14,15], Alexandros C. Dimopoulos[19], Sosipatros A. Boikos[20], Angeliki M. Stamatouli[21], Janina P. Lewis[1,22,23], Masoud H. Manjili[23], Hiromichi Ebi[12,24], Kristoffer Valerie[25], Renfeng Li[16,17,18], Andrew Poklepovic[26], Jennifer E. Koblinski[6], Trevor Siggers[7,8,27], Ana Banito[9,10], Mikhail G. Dozmorov[28], Kevin B. Jones[4]*, Senthil K. Radhakrishnan[6]* and Anthony C. Faber[1,2]**

[1]VCU Philips Institute, Virginia Commonwealth University School of Dentistry and Massey Comprehensive Cancer Center, Richmond VA, 23298 USA.
[2]Department of Pediatrics, Virginia Commonwealth University School of Medicine, Richmond, VA, 23298 USA.
[3]Pauley Heart Center, Division of Cardiology, Department of Internal Medicine, Virginia Commonwealth University, Richmond, Virginia, USA.
[4]Departments of Orthopaedics and Oncological Sciences, Huntsman Cancer Institute, University of Utah, 2000 Circle of Hope Drive, Salt Lake City, UT 84112 USA.
[5]Division of Oncology, Department of Internal Medicine, Washington University School of Medicine Saint Louis, MO 63110 USA.
[6]Department of Pathology, Virginia Commonwealth University and Massey Comprehensive Cancer Center, Richmond VA, 23298 USA.
[7]Department of Biology, Boston University, Boston, MA 02215, USA.
[8]Biological Design Center, Boston University, Boston, MA 02215, USA.
[9]Soft Tissue Sarcoma Research Group, Hopp Children’s Cancer Center, Heidelberg (KiTZ), German Cancer Research Center (DKFZ), Heidelberg, Germany.
[10]National Center for Tumor Diseases (NCT), NCT Heidelberg, A Partnership between DKFZ and Heidelberg University Hospital, Heidelberg, Germany.
[11]Faculty of Biosciences, University of Heidelberg, 69120 Heidelberg, Germany.
[12]Renal Section, Department of Medicine, Boston Medical Center, Boston University School of Medicine, Boston, Massachusetts, USA.
[13]Division of Molecular Therapeutics, Aichi Cancer Center Research Institute, Nagoya, Japan.
[14]Greehey Children's Cancer Research Institute, University of Texas Health San Antonio, San Antonio, Texas, USA.
[15]Department of Molecular Medicine, University of Texas Health San Antonio, San Antonio, Texas, USA.
[16]Program in Microbiology and Immunology, University of Pittsburgh, Pittsburgh, Pennsylvania, USA.
[17]Department of Microbiology and Molecular Genetics, University of Pittsburgh, Pittsburgh, Pennsylvania, USA.
[18]Cancer Virology Program, Hillman Cancer Center, University of Pittsburgh Medical Center, Pittsburgh, PA 15232, USA.

[19]Department of Informatics and Telematics, School of Digital Technology, Harokopio University, Athens, Greece.
[20]Georgetown Lombardi Comprehensive Cancer Center, 3800 Reservoir Rd NW Ste E501, Washington, DC 20007 USA.
[21]Division of Endocrinology, Diabetes, and Metabolism, Department of Internal Medicine, Virginia Commonwealth University School of Medicine, Richmond, VA, 23298, USA.
[22]Department of Biochemistry and Molecular Biology, and Massey Comprehensive Cancer Center, Virginia Commonwealth University, Richmond, VA, 23298, USA.
[23]Department of Microbiology & Immunology and Massey Comprehensive Cancer Center, Richmond VA, 23298, USA.
[24]Division of Advanced Cancer Therapeutics, Nagoya University Graduate School of Medicine, Nagoya, Aichi, Japan.
[25]Department of Radiation Oncology and Massey Comprehensive Cancer Center, Virginia Commonwealth University, Richmond VA, 23298 USA.
[26]Department of Internal Medicine, Division of Oncology, Massey Comprehensive Cancer Center, Virginia Commonwealth University, Richmond, VA, 23298, USA.
[27]Bioinformatics Program, Boston University, Boston, MA 02215, USA.
[28]Department of Biostatistics, Virginia Commonwealth University, Richmond VA, 23298, USA.

# equal contribution
*co-corresponding author
**correspondence: acfaber@vcu.edu

Table of contents:
Supp. Figures 1-10

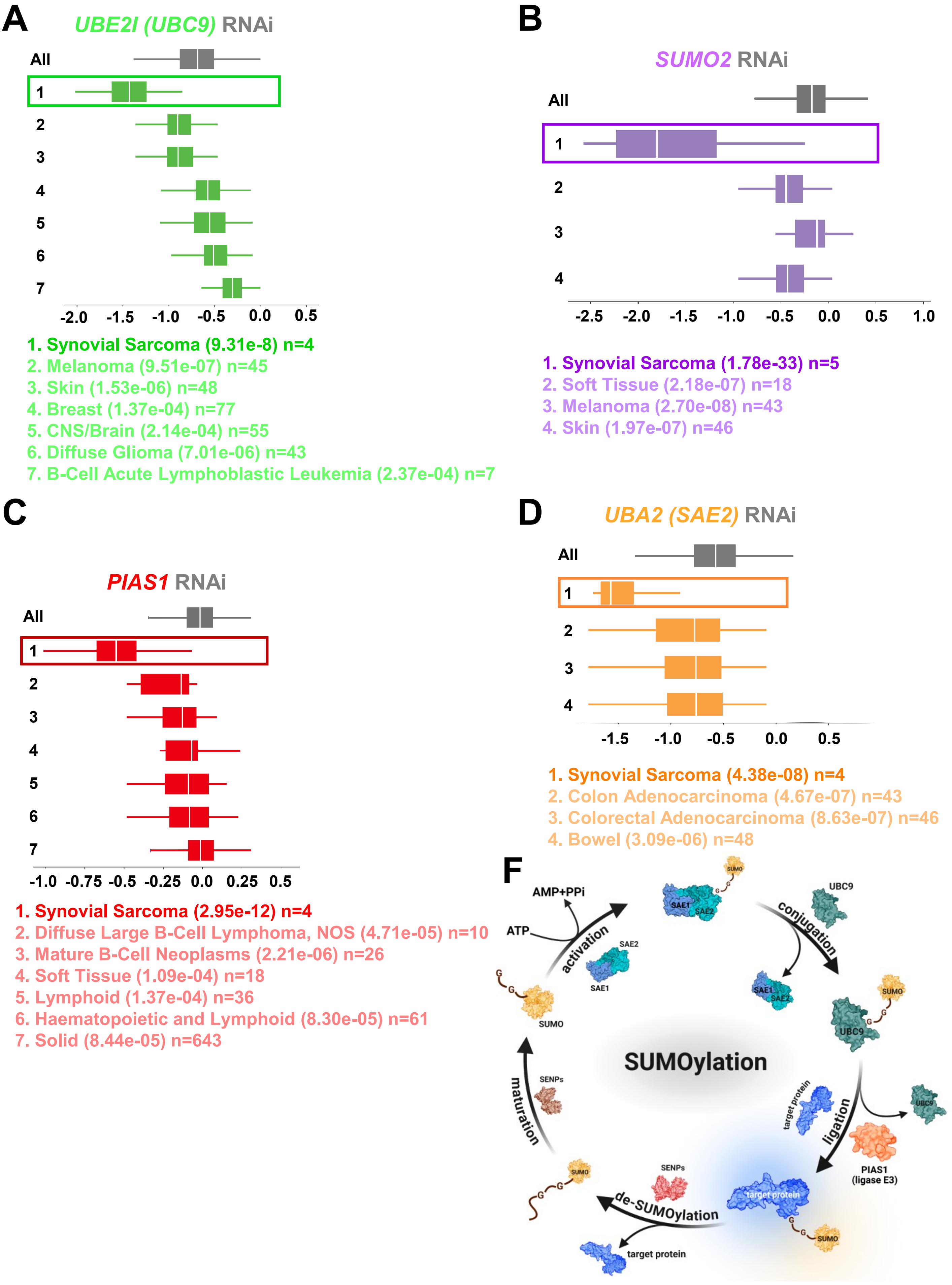
A
UBE2I (UBC9) RNAi
All
1
2
3
4
5
6
7
-2.0 -1.5 -1.0 -0.5 0.0 0.5
1. Synovial Sarcoma (9.31e-8) n=4
2. Melanoma (9.51e-07) n=45
3. Skin (1.53e-06) n=48
4. Breast (1.37e-04) n=77
5. CNS/Brain (2.14e-04) n=55
6. Diffuse Glioma (7.01e-06) n=43
7. B-Cell Acute Lymphoblastic Leukemia (2.37e-04) n=7
B
SUMO2 RNAi
All
1
2
3
4
-2.5 -2.0 -1.5 -1.0 -0.5 0.0 0.5 1.0
1. Synovial Sarcoma (1.78e-33) n=5
2. Soft Tissue (2.18e-07) n=18
3. Melanoma (2.70e-08) n=43
4. Skin (1.97e-07) n=46
C
PIAS1 RNAi
All
1
2
3
4
5
6
7
-1.0 -0.75 -0.5 -0.25 0.0 0.25 0.5
1. Synovial Sarcoma (2.95e-12) n=4
2. Diffuse Large B-Cell Lymphoma, NOS (4.71e-05) n=10
3. Mature B-Cell Neoplasms (2.21e-06) n=26
4. Soft Tissue (1.09e-04) n=18
5. Lymphoid (1.37e-04) n=36
6. Haematopoietic and Lymphoid (8.30e-05) n=61
7. Solid (8.44e-05) n=643
D
UBA2 (SAE2) RNAi
All
1
2
3
4
-1.5 -1.0 -0.5 0.0 0.5
1. Synovial Sarcoma (4.38e-08) n=4
2. Colon Adenocarcinoma (4.67e-07) n=43
3. Colorectal Adenocarcinoma (8.63e-07) n=46
4. Bowel (3.09e-06) n=48
F
AMP+PPi
ATP
activation
SAE1
SAE2
SUMO
UBC9
conjugation
SUMOylation
target protein
ligation
PIAS1
(ligase E3)
SENPs
de-SUMOylation
maturation

Supp. Figure 1. Synovial sarcoma is the most sensitive subtype of cancer to knocking down the main SUMO components. A) - E) DepMap (Dependency map) consortium genomic screen data in four SS cell lines represented among the ~700 cancer cell lines. In this analysis from RNAi screens (Achilles + DRIVE + Marcotte), we noted sensitivity of siRNAs targeting several members of the SUMOylation pathway (*UBE2I* (*UBC9*), *SUMO2*, *PIAS1* and *UBA2* (*SAE2*)), as analyzed by the Broad Institute scoring algorithm, DEMETER2. **F)** Schema of SUMOylation (Created in BioRender. Floros, K. (2025).

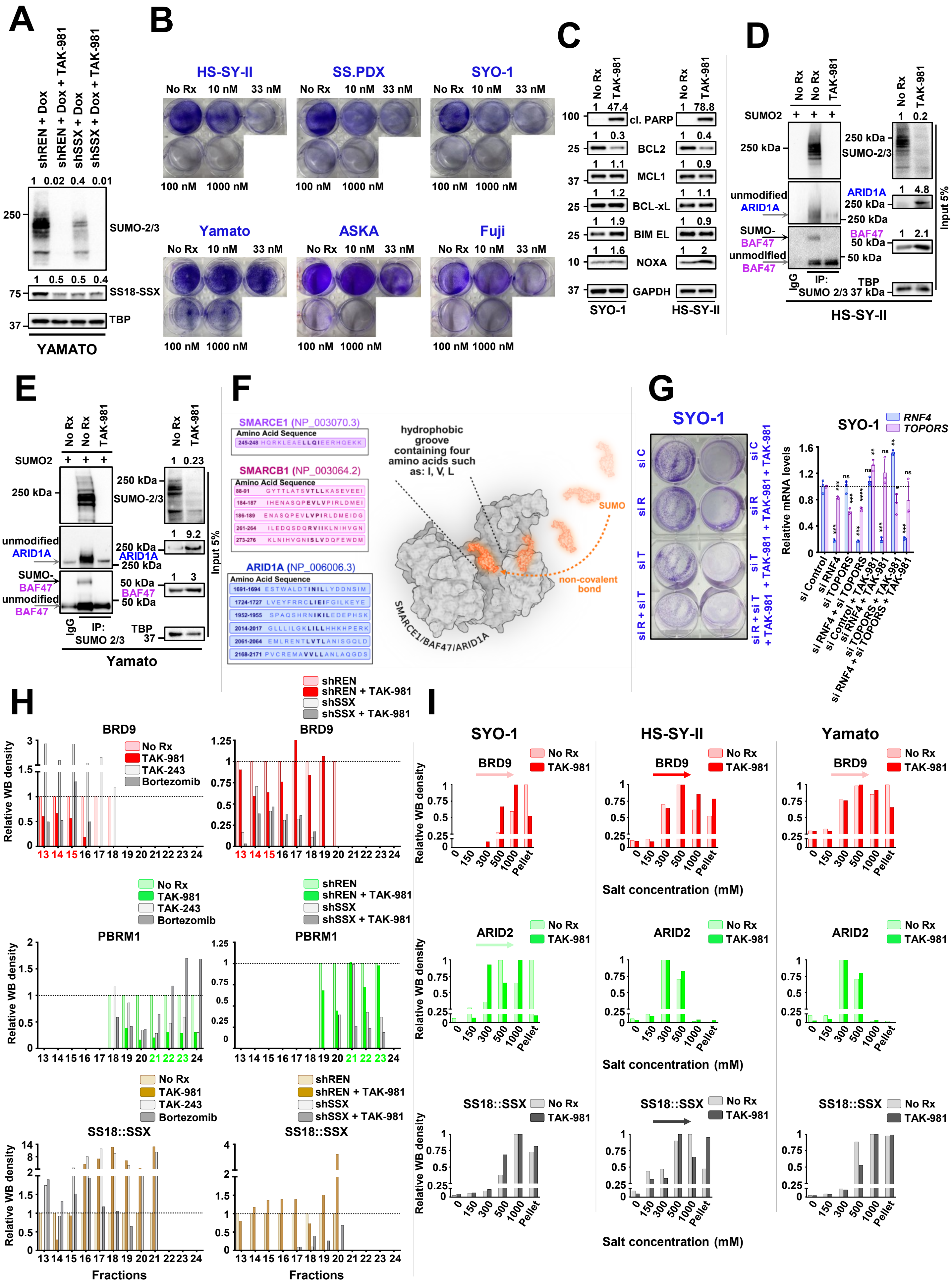
A
shREN + Dox
shREN + Dox + TAK-981
shSSX + Dox
shSSX + Dox + TAK-981
1 0.02 0.4 0.01
250
SUMO-2/3
1 0.5 0.5 0.4
75
SS18-SSX
37
TBP
YAMATO
B
HS-SY-II
SS.PDX
SYO-1
Yamato
ASKA
Fuji
No Rx 10 nM 33 nM
100 nM 1000 nM
C
No Rx
TAK-981
1 47.4
1 78.8
cl. PARP
1 0.3
1 0.4
BCL2
1 1.1
1 0.9
MCL1
1 1.2
1 1.1
BCL-xL
1 1.9
1 0.9
BIM EL
1 1.6
1 2
NOXA
GAPDH
SYO-1
HS-SY-II
D
SUMO2
250 kDa
SUMO-2/3
unmodified
ARID1A
SUMO-
BAF47
unmodified
BAF47
IgG
IP:
SUMO 2/3
1 0.2
1 4.8
1 2.1
50 kDa
TBP
37 kDa
Input 5%
HS-SY-II
E
SUMO2
250 kDa
SUMO-2/3
1 0.23
1 9.2
1 3
unmodified
ARID1A
SUMO-
BAF47
unmodified
BAF47
IgG
IP:
SUMO 2/3
TBP
Input 5%
Yamato
F
SMARCE1 (NP_003070.3)
Amino Acid Sequence
245-248 HQRKLEAELLQIEERHQEKK
SMARCB1 (NP_003064.2)
Amino Acid Sequence
88-91 GYTTLATSVTLLKASEVEEI
184-187 IHENASQPEVLVPIRLDMEI
186-189 ENASQPEVLVPIRLDMEIDG
261-264 ILEDQSDQRVIIKLNIHVGN
273-276 KLNIHVGNISLVDQFEWDM
ARID1A (NP_006006.3)
Amino Acid Sequence
1691-1694 ESTWALDTINILLYDDNSIM
1724-1727 LVEYFRRCLIEIFGILKEYE
1952-1955 SPAQSHRNIKILEDEPHSK
2014-2017 GLLLILGKLILLHHKHPERK
2061-2064 EMLRENTLVTLANISGQLD
2168-2171 PVCREMAVVLLANLAQGDS
hydrophobic groove containing four amino acids such as: I, V, L
SUMO
non-covalent bond
SMARCE1/BAF47/ARID1A
G
SYO-1
si C
si R
si T
si R + si T
si C + TAK-981
si R + TAK-981
si T + TAK-981
si R + si T + TAK-981
RNF4
TOPORS
Relative mRNA levels
si Control
si RNF4
si TOPORS
si RNF4 + si TOPORS
si Control + TAK-981
si RNF4 + TAK-981
si TOPORS + TAK-981
si RNF4 + si TOPORS + TAK-981
H
BRD9
PBRM1
SS18::SSX
No Rx
TAK-981
TAK-243
Bortezomib
shREN
shREN + TAK-981
shSSX
shSSX + TAK-981
Relative WB density
13 14 15 16 17 18 19 20 21 22 23 24
Fractions
I
SYO-1
HS-SY-II
Yamato
BRD9
ARID2
SS18::SSX
No Rx
TAK-981
Relative WB density
0 150 300 500 1000 Pellet
Salt concentration (mM)

Supp. Figure 2. Knockdown of SS18::SSX results in attenuation of the SUMOylation program. SUMO inhibition leads to SS cell death through stabilization of the cBAF complex. A) Yamato SS cells were infected with a doxycycline (DOX)-inducible control (shREN) or SS18::SSX knockdown (shSSX) and treated with 100nM TAK-981 for 36h and nuclear lysates were probed with the indicated antibodies. Protein band intensities were quantified as in the main figures. **B)** Crystal violet assays of SS cells untreated (No Rx) or treated with increasing concentrations of TAK-981 for five days. **C)** SS cells were treated with 100nM TAK-981 for 36h or left untreated (No Rx) and whole cell lysates were probed with the indicated antibodies. **D)** and **E)** Like in Fig. 2B and 2D, here for SMARCE1, SUMOylation of BAF47 and ARID1A was investigated. Supp. Figs. 2D and 2E share the same western blots for SUMO-2/3 and TBP (loading control) with Figs. 2B and 2D. **F)** The potential SUMO interacting motifs (SIMs) of ARID1A, BAF47 and SMARCE1 were detected by JASSA analyzer[1] (Created in BioRender. Floros, K. (2025)). **G left)** SYO-1 cells were transfected with siControl, siRNF4, siTOPORS and the combination of siRNF4 and siTOPORS for 48h, following treatment with no drug or 100 nM of TAK-981 for 5d and stained with crystal violet. **G right)** The abundance of *RNF4* and *TOPORS* were analyzed by qPCR in SYO-1 cells, like what was conducted for HS-SY-II and SYO-1 in Fig. 2G and 2**I.** Statistical analysis was performed as in Fig. 2G and 2I. **H)** Band intensities for the specific proteins were quantified as in the main figures and normalized for each fraction to the value of No Rx (S2H, left) and shREN (S2H, right) for each BAF complex subunit separately. A line was added to the No Rx or the shREN WB relative density levels (''1'') to simplify visual comparisons. **I)** The band densities of BRD9, ARID2 and SS18::SSX for the three SS cell lines from Fig. 4A were graphed using the GraphPad Prism software. Exact *p*-values and source Data can be found in the Source Data.

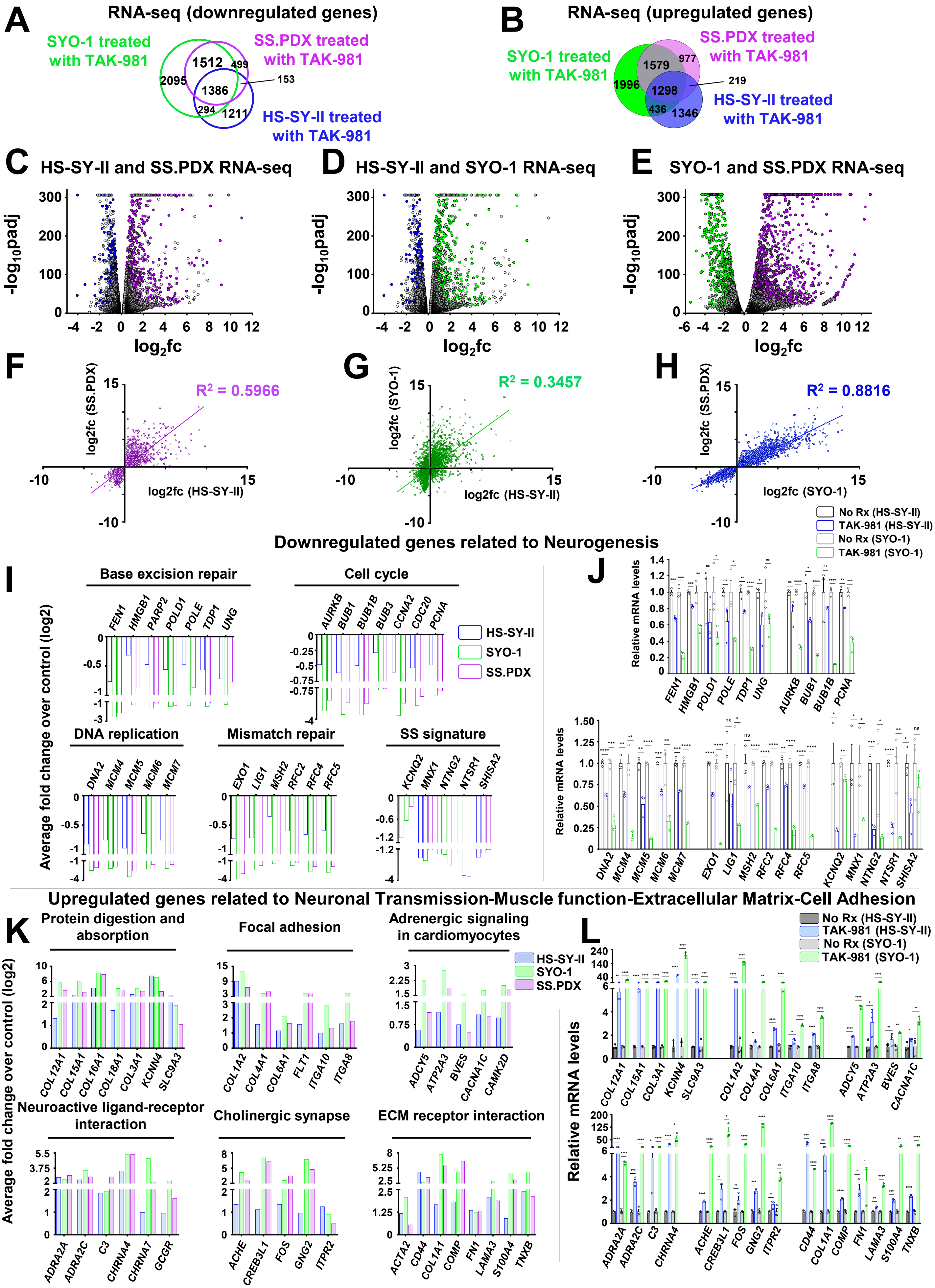

A
RNA-seq (downregulated genes)
SYO-1 treated with TAK-981
SS.PDX treated with TAK-981
HS-SY-II treated with TAK-981
B
RNA-seq (upregulated genes)
SYO-1 treated with TAK-981
SS.PDX treated with TAK-981
HS-SY-II treated with TAK-981
C
HS-SY-II and SS.PDX RNA-seq
D
HS-SY-II and SYO-1 RNA-seq
E
SYO-1 and SS.PDX RNA-seq
F
$R^2 = 0.5966$
G
$R^2 = 0.3457$
H
$R^2 = 0.8816$
No Rx (HS-SY-II)
TAK-981 (HS-SY-II)
No Rx (SYO-1)
TAK-981 (SYO-1)
Downregulated genes related to Neurogenesis
I
Base excision repair
Cell cycle
DNA replication
Mismatch repair
SS signature
Average fold change over control (log2)
J
Relative mRNA levels
Upregulated genes related to Neuronal Transmission-Muscle function-Extracellular Matrix-Cell Adhesion
K
Protein digestion and absorption
Focal adhesion
Adrenergic signaling in cardiomyocytes
Neuroactive ligand-receptor interaction
Cholinergic synapse
ECM receptor interaction
L
Relative mRNA levels

Supp. Figure 3. Blocking of SUMOylation reduces the transcriptome that contains genes related to neurogenesis and rewires the cells towards a mesenchymal phenotype. A) Venn diagram of downregulated genes from RNA-seq following 100nM TAK-981 treatment for 36h in three SS cell lines from Fig. 5. **B)** Venn diagram of upregulated genes from RNA-seq following 100nM TAK-981 treatment for 36h in three SS cell lines from Fig. 5. **C) - E)** Volcano plots for pairwise comparisons between the three SS cell lines from RNA-seq following 100nM TAK-981 treatment for 36h from Fig. 5. The *p*-values were adjusted using a False Discovery Rate (FDR) multiple testing correction method. **F) - H)** Linear Regression analysis from pairwise comparisons between the three SS cell lines from RNA-seq following 100nM TAK-981 treatment for 36h. **I)** Plotted RNA-Seq fold changes of genes downregulated after TAK-981 treatment and pathway analysis. **J)** Quantitative RT-PCR validating expression results of genes obtained by RNA-Seq for downregulated genes from (I). n = 3 biological replicates; error bars indicate +SEM. Unpaired two-tailed t tests were performed for all comparisons between No Rx and TAK-981 for both HS-SY-II and SYO-1. **K)** Plotted RNA-Seq fold changes of genes upregulated after TAK-981 treatment and pathway analysis. **L)** qPCR validation of genes from (K). n = 3 biological replicates; error bars indicate +SEM. Unpaired two-sided t test was performed for all comparisons between No Rx and TAK-981 for each cell line and each gene separately. Exact *p*-values and source Data can be found in the Source Data.

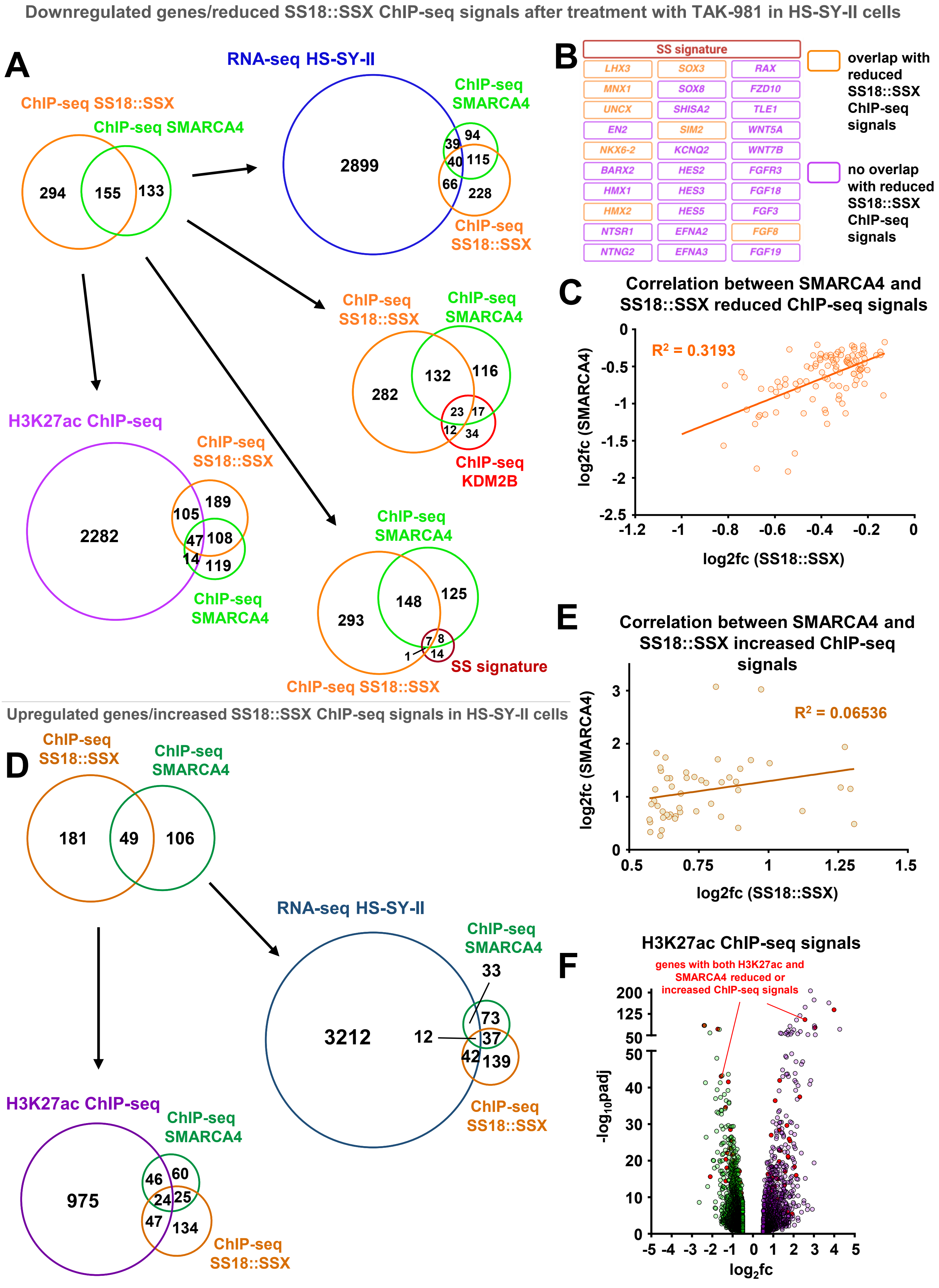

Downregulated genes/reduced SS18::SSX ChIP-seq signals after treatment with TAK-981 in HS-SY-II cells
A
ChIP-seq SS18::SSX
ChIP-seq SMARCA4
294
155
133
RNA-seq HS-SY-II
2899
ChIP-seq SMARCA4
39
94
40
115
66
228
ChIP-seq SS18::SSX
ChIP-seq SS18::SSX
ChIP-seq SMARCA4
282
132
116
23
17
12
34
ChIP-seq KDM2B
H3K27ac ChIP-seq
ChIP-seq SS18::SSX
2282
189
105
47
108
14
119
ChIP-seq SMARCA4
ChIP-seq SMARCA4
293
148
125
7
8
1
14
SS signature
ChIP-seq SS18::SSX
B
SS signature
LHX3
SOX3
RAX
MNX1
SOX8
FZD10
UNCX
SHISA2
TLE1
EN2
SIM2
WNT5A
NKX6-2
KCNQ2
WNT7B
BARX2
HES2
FGFR3
HMX1
HES3
FGF18
HMX2
HES5
FGF3
NTSR1
EFNA2
FGF8
NTNG2
EFNA3
FGF19
overlap with reduced SS18::SSX ChIP-seq signals
no overlap with reduced SS18::SSX ChIP-seq signals
C
Correlation between SMARCA4 and SS18::SSX reduced ChIP-seq signals
R² = 0.3193
log2fc (SMARCA4)
log2fc (SS18::SSX)
E
Correlation between SMARCA4 and SS18::SSX increased ChIP-seq signals
R² = 0.06536
log2fc (SMARCA4)
log2fc (SS18::SSX)
Upregulated genes/increased SS18::SSX ChIP-seq signals in HS-SY-II cells
D
ChIP-seq SS18::SSX
ChIP-seq SMARCA4
181
49
106
RNA-seq HS-SY-II
ChIP-seq SMARCA4
3212
33
12
73
37
42
139
ChIP-seq SS18::SSX
H3K27ac ChIP-seq
ChIP-seq SMARCA4
975
46
60
24
25
47
134
ChIP-seq SS18::SSX
F
H3K27ac ChIP-seq signals
genes with both H3K27ac and SMARCA4 reduced or increased ChIP-seq signals
-log10padj
log2fc

Supp. Figure 4. The binding of BAF complexes to chromatin is responsible for many of the changes of the SS transcriptome after blocking SUMOylation in HS-SY-II cells. A) Venn diagrams of HS-SY-II cells treated with 100nM TAK-981 for 36h (and compared to untreated HS-SY-II cells) and significantly downregulated signals from ChIP-seq experiments (including SS18::SSX, SMARCA4, KDM2B and H3K27ac) and/or RNA-seq from our study and/or the SS signature. **B)** The genes from the SS signature and those that overlap with reduced SS18::SSX ChIP-seq signals (orange boxes) or that do not (purple boxes) in HS-SY-II cells (Created in BioRender. Floros, K. (2025)). **C)** Linear regression analysis of the correlation between SMARCA4 and SS18::SSX reduced ChIP-seq signals in HS-SY-II cells. **D)** Venn diagrams of HS-SY-II cells treated with 100nM TAK-981 for 36h (and compared to untreated HS-SY-II cells) and significantly upregulated signals from ChIP-seq experiments (including SS18::SSX, SMARCA4 and H3K27ac) and/or RNA-seq analysis from our study. **E)** Linear regression analysis of the correlation between SMARCA4 and SS18::SSX increased ChIP-seq signals in HS-SY-II cells. **F)** Volcano plot illustrates the ChIP-seq profile of reduced (green) and increased (purple) H3K27ac signals after TAK-981 treatment in HS-SY-II cells. Red dots represent the genes with commonly reduced or increased H3K27ac and SMARCA4 ChIP-seq signals. For Supp. Figure 4F the *p*-values were adjusted using a False Discovery Rate (FDR) multiple testing correction method. padj: adjusted *p*-value.

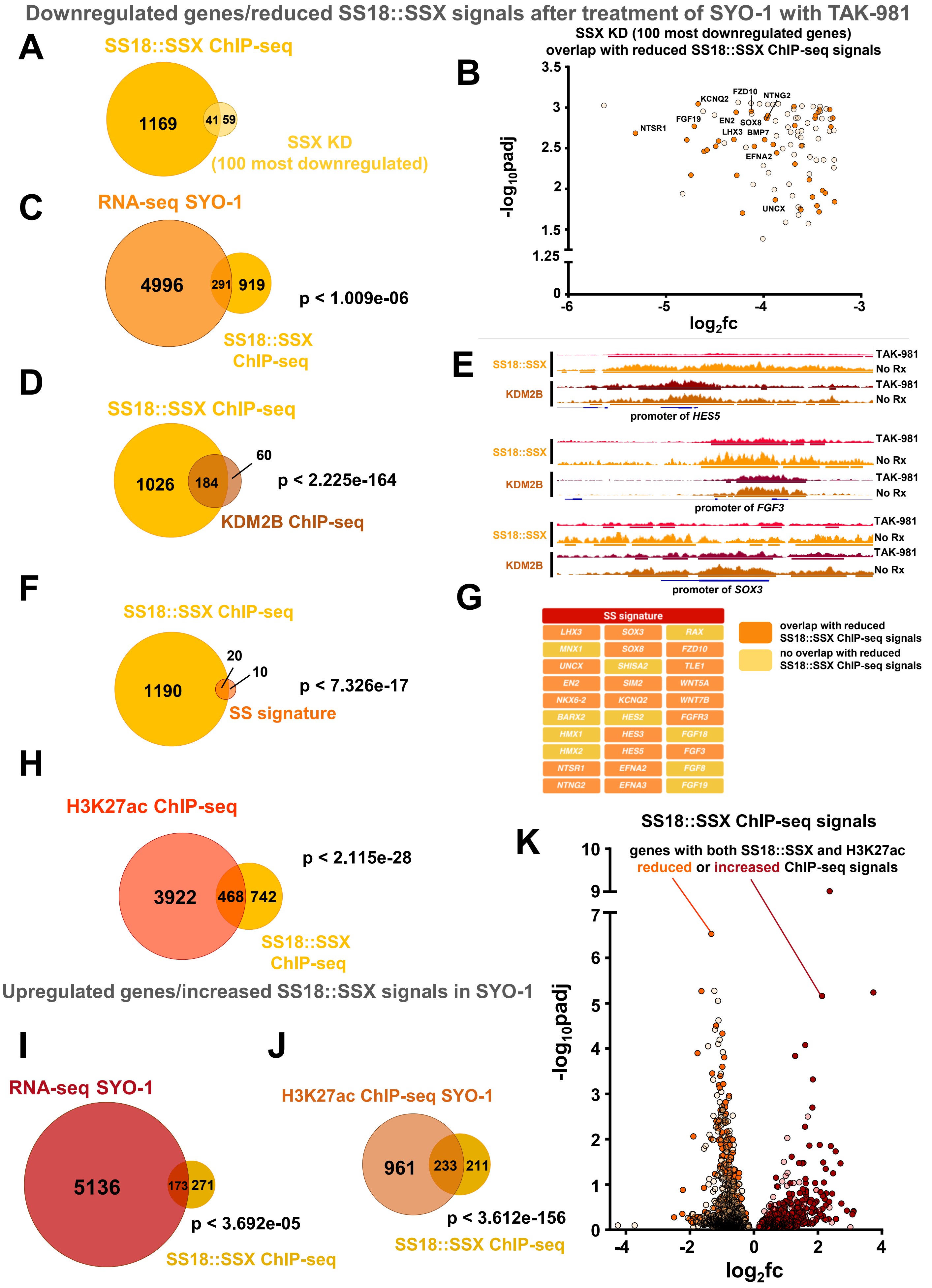
Downregulated genes/reduced SS18::SSX signals after treatment of SYO-1 with TAK-981
A
SS18::SSX ChIP-seq
1169
41
59
SSX KD
(100 most downregulated)
B
SSX KD (100 most downregulated genes)
overlap with reduced SS18::SSX ChIP-seq signals
-log10padj
log2fc
NTSR1
KCNQ2
FZD10
NTNG2
FGF19
EN2
SOX8
LHX3
BMP7
EFNA2
UNCX
C
RNA-seq SYO-1
4996
291
919
p < 1.009e-06
SS18::SSX
ChIP-seq
D
SS18::SSX ChIP-seq
1026
184
60
p < 2.225e-164
KDM2B ChIP-seq
E
SS18::SSX
KDM2B
TAK-981
No Rx
promoter of HES5
promoter of FGF3
promoter of SOX3
F
SS18::SSX ChIP-seq
1190
20
10
p < 7.326e-17
SS signature
G
SS signature
LHX3
SOX3
RAX
MNX1
SOX8
FZD10
UNCX
SHISA2
TLE1
EN2
SIM2
WNT5A
NKX6-2
KCNQ2
WNT7B
BARX2
HES2
FGFR3
HMX1
HES3
FGF18
HMX2
HES5
FGF3
NTSR1
EFNA2
FGF8
NTNG2
EFNA3
FGF19
overlap with reduced
SS18::SSX ChIP-seq signals
no overlap with reduced
SS18::SSX ChIP-seq signals
H
H3K27ac ChIP-seq
3922
468
742
p < 2.115e-28
SS18::SSX
ChIP-seq
Upregulated genes/increased SS18::SSX signals in SYO-1
I
RNA-seq SYO-1
5136
173
271
p < 3.692e-05
SS18::SSX ChIP-seq
J
H3K27ac ChIP-seq SYO-1
961
233
211
p < 3.612e-156
SS18::SSX ChIP-seq
K
SS18::SSX ChIP-seq signals
genes with both SS18::SSX and H3K27ac
reduced or increased ChIP-seq signals
-log10padj
log2fc

Supp. Figure 5. Pharmacological inhibition of SUMOylation in SYO-1 cells elicits changes in the ChIP-seq signals similar to the changes in gene expression following SS18::SSX knockdown. A) Venn diagram of SYO-1 cells treated with 100nM TAK-981 for 36h (and compared to untreated SYO-1 cells) and significantly downregulated signals from SS18::SSX ChIP-seq compared to the 100 most downregulated genes after SS18::SSX knockdown in HS-SY-II cells (conducted by Banito et al.[2]). **B)** Genes that overlap from (A) are plotted (orange dots indicate overlapping genes). **C)** Venn diagram of significantly downregulated genes from the RNA-seq experiment (conducted in the current study) compared to significantly downregulated signals from the SS18::SSX ChIP-seq experiment. **D)** Venn diagram of significantly downregulated signals from the SS18::SSX ChIP-seq experiment compared to significantly downregulated signals from the KDM2B ChIP-seq experiment in SYO-1 cells treated as in A). **E)** Depth-normalized SS18::SSX and KDM2B ChIP-seq tracks using IGV genome browser, reflecting losses in SS18::SSX and KDM2B peaks at the locus of genes supporting synovial sarcomagenesis in the SYO-1 cells. **F)** Venn diagram of significantly downregulated signals from the SS18::SSX ChIP-seq experiment compared to significantly downregulated signals from the SS signature. **G)** The genes from the SS signature and those that overlap with reduced SS18::SSX ChIP-seq signals (orange boxes) or that do not (yellow boxes) after TAK-981 treatment in SYO-1 cells. **H)** Venn diagram of significantly downregulated signals from the H3K27ac ChIP-seq experiment compared to significantly downregulated signals from the SS18::SSX ChIP-seq experiment. **I)** Venn diagram of significantly upregulated genes from our RNA-seq data (Fig. 5) compared to significantly increased signals from the SS18::SSX ChIP-seq experiment. **J)** Venn diagram of significantly upregulated genes from the H3K27ac ChIP-seq experiment compared to significantly reduced signals from the SS18::SSX ChIP-seq experiment. **K)** Volcano plot that illustrates the ChIP-seq profile of reduced (orange) and increased (red) SS18::SSX signals after TAK-981 treatment in SYO-1 cells. For Supp. Fig. 5B and Supp. Fig. 5K the *p*-values were adjusted using a False Discovery Rate (FDR) multiple testing correction method. The table of Supp. Fig. 5G was created in BioRender. Floros, K. (2025).

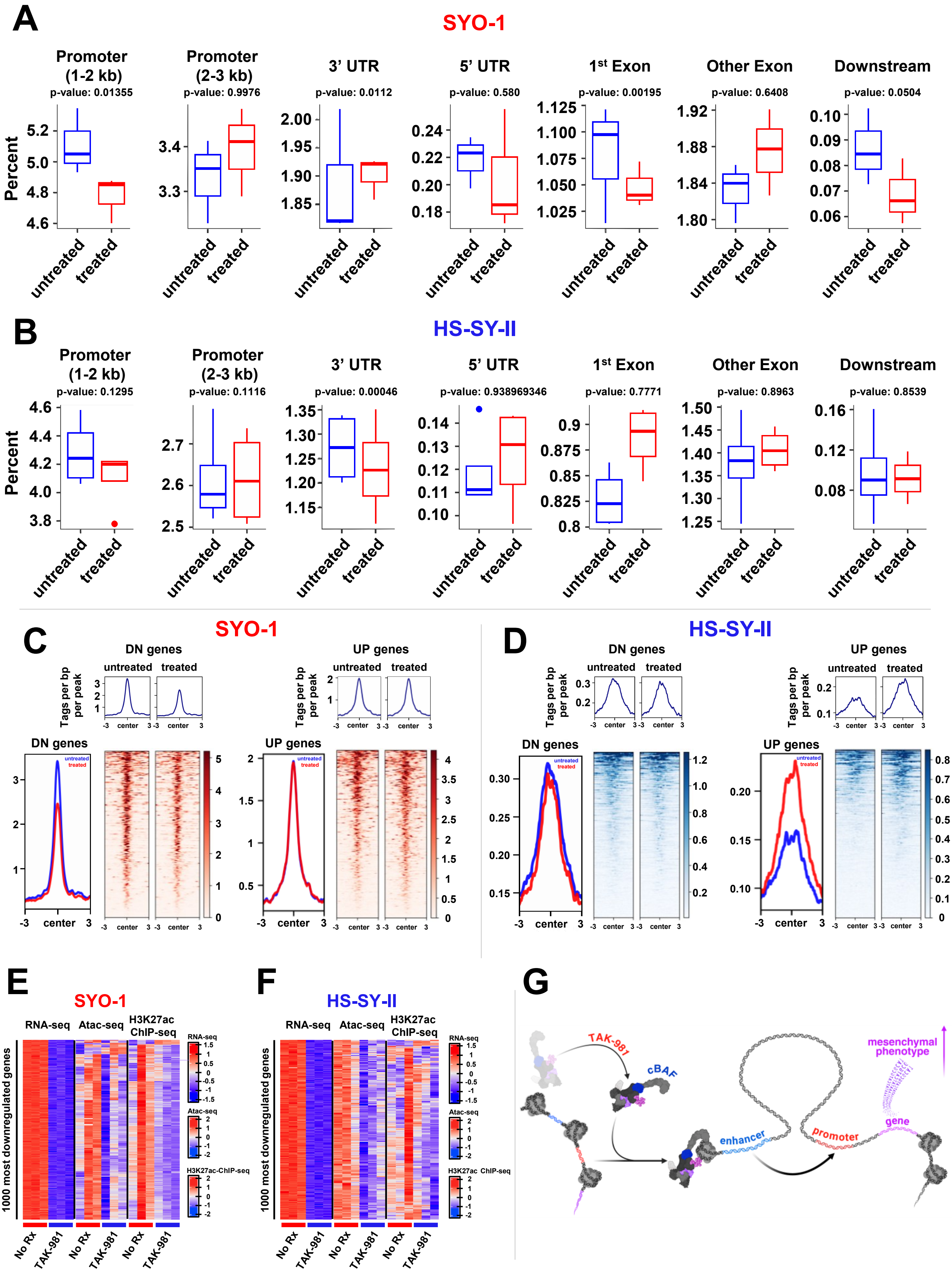
A
SYO-1
Promoter (1-2 kb)
p-value: 0.01355
Promoter (2-3 kb)
p-value: 0.9976
3' UTR
p-value: 0.0112
5' UTR
p-value: 0.580
1st Exon
p-value: 0.00195
Other Exon
p-value: 0.6408
Downstream
p-value: 0.0504
Percent
untreated
treated
B
HS-SY-II
Promoter (1-2 kb)
p-value: 0.1295
Promoter (2-3 kb)
p-value: 0.1116
3' UTR
p-value: 0.00046
5' UTR
p-value: 0.938969346
1st Exon
p-value: 0.7771
Other Exon
p-value: 0.8963
Downstream
p-value: 0.8539
C
SYO-1
DN genes
UP genes
Tags per bp per peak
center
D
HS-SY-II
E
SYO-1
RNA-seq
Atac-seq
H3K27ac ChIP-seq
1000 most downregulated genes
No Rx
TAK-981
F
HS-SY-II
G
TAK-981
cBAF
enhancer
promoter
gene
mesenchymal phenotype

Supp. Figure 6. Addition of TAK-981 leads to enhancement of chromatin accessibility at introns and at distal intergenic sites of the genes that promote synovial sarcomagenesis. A) and **B)** Boxplots demonstrating distribution of the rest of the SYO-1 and HS-SY-II ATAC-seq peaks genome-wide (from Fig. 7A and 7B), focusing on downregulated RNA-Seq normalized genes for HS-SY-II cells transduced with SS18::SSX shRNA from Banito et al.[2] (filtered like in Figure 7A and 7B). UTR, untranslated region. Differential accessibility peaks were annotated using the ChIPseeker v.1.40.0 R package and percentages of peaks associated with various gene-centric annotations were compared using unpaired two-sided *t*-test. The boxplots show the median (central line), and the interquartile range (the range between the first quartile (25th percentile) and the third quartile (75th percentile) of the data). The whiskers extend to the minimum and to the maximum values excluding outliers. **C)** and **D)** Average tag density profiles (top) and heatmap plots of ATAC-seq signal centered on upregulated (left) and downregulated (right) Lowe's genes for untreated (blue) and treated (red) samples. Heatmaps show signal intensity for individual genes-associated peaks, sorted by peak strength. **E)** and **F)** Heatmaps of jointly clustered row-normalized gene expression (RNA-seq), and signal (ATAC-seq, ChIP-seq) of the top 1000 most downregulated genes and the associated peaks before and after TAK-981 treatment (number of replicates for each condition, n = 3). **G)** Graphical depiction of our suggested model supporting an increase in expression of genes related to the mesenchymal phenotype, following augmented chromatin accessibility and enhancer activation caused by elevated cBAF occupancy after TAK-981 treatment (Created in BioRender. Floros, K. (2025)).

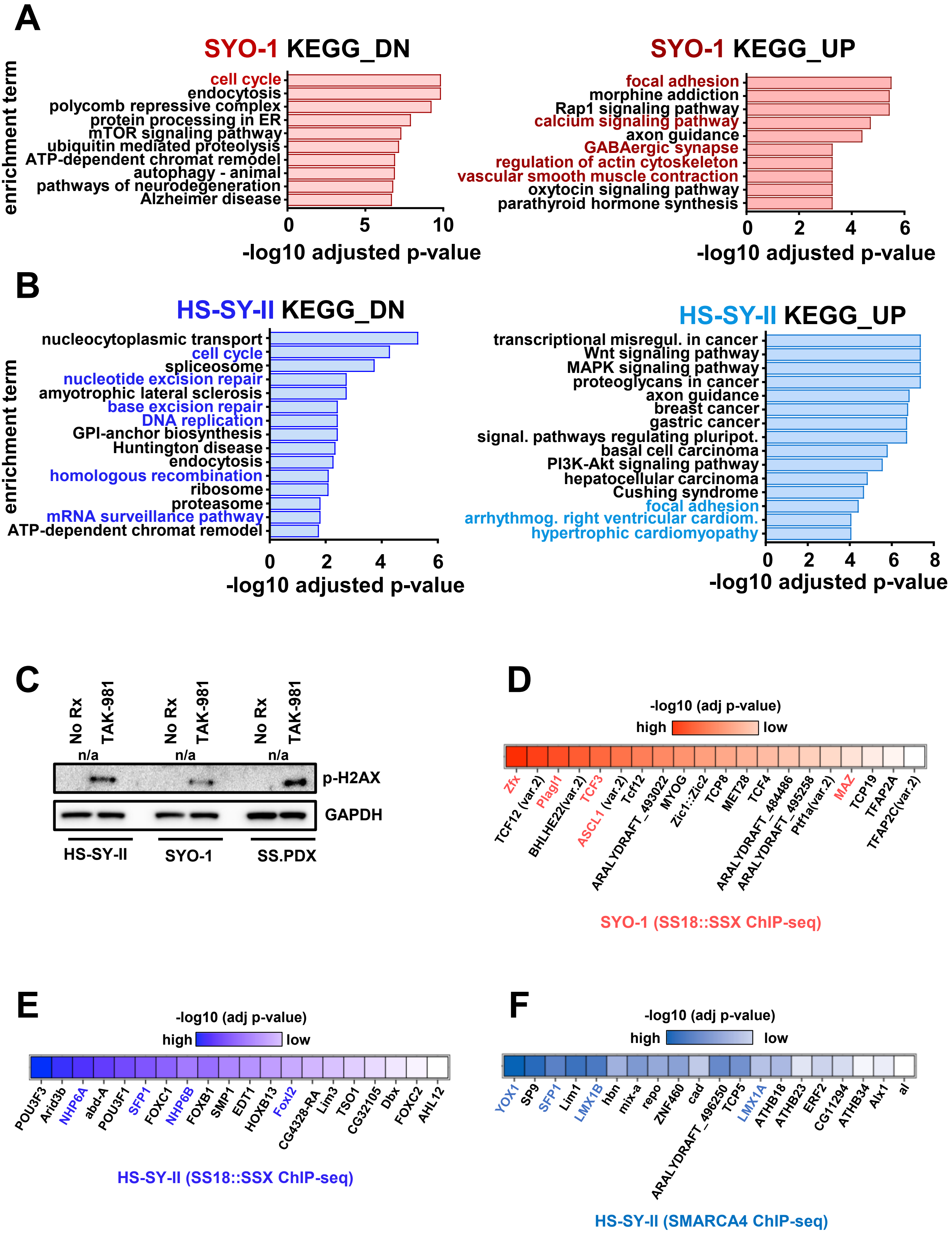
A
SYO-1 KEGG_DN
cell cycle
endocytosis
polycomb repressive complex
protein processing in ER
mTOR signaling pathway
ubiquitin mediated proteolysis
ATP-dependent chromat remodel
autophagy - animal
pathways of neurodegeneration
Alzheimer disease
enrichment term
-log10 adjusted p-value
SYO-1 KEGG_UP
focal adhesion
morphine addiction
Rap1 signaling pathway
calcium signaling pathway
axon guidance
GABAergic synapse
regulation of actin cytoskeleton
vascular smooth muscle contraction
oxytocin signaling pathway
parathyroid hormone synthesis
-log10 adjusted p-value
B
HS-SY-II KEGG_DN
nucleocytoplasmic transport
cell cycle
spliceosome
nucleotide excision repair
amyotrophic lateral sclerosis
base excision repair
DNA replication
GPI-anchor biosynthesis
Huntington disease
endocytosis
homologous recombination
ribosome
proteasome
mRNA surveillance pathway
ATP-dependent chromat remodel
enrichment term
-log10 adjusted p-value
HS-SY-II KEGG_UP
transcriptional misregul. in cancer
Wnt signaling pathway
MAPK signaling pathway
proteoglycans in cancer
axon guidance
breast cancer
gastric cancer
signal. pathways regulating pluripot.
basal cell carcinoma
PI3K-Akt signaling pathway
hepatocellular carcinoma
Cushing syndrome
focal adhesion
arrhythmog. right ventricular cardiom.
hypertrophic cardiomyopathy
-log10 adjusted p-value
C
No Rx
TAK-981
n/a
p-H2AX
GAPDH
HS-SY-II
SYO-1
SS.PDX
D
-log10 (adj p-value)
high
low
Zfx
TCF12 (var.2)
Plagl1
BHLHE22(var.2)
TCF3
ASCL1 (var.2)
Tcf12
ARALYDRAFT_493022
MYOG
Zic1::Zic2
TCP8
MET28
TCF4
ARALYDRAFT_484486
ARALYDRAFT_495258
Ptf1a(var.2)
MAZ
TCP19
TFAP2A
TFAP2C(var.2)
SYO-1 (SS18::SSX ChIP-seq)
E
-log10 (adj p-value)
high
low
POU3F3
Arid3b
NHP6A
abd-A
POU3F1
SFP1
FOXC1
NHP6B
FOXB1
SMP1
EDT1
HOXB13
Foxl2
CG4328-RA
Lim3
TSO1
CG32105
Dbx
FOXC2
AHL12
HS-SY-II (SS18::SSX ChIP-seq)
F
-log10 (adj p-value)
high
low
YOX1
SP9
SFP1
Lim1
LMX1B
hbn
mix-a
repo
ZNF460
cad
ARALYDRAFT_496250
TCP5
LMX1A
ATHB18
ATHB23
ERF2
CG11294
ATHB34
Alx1
al
HS-SY-II (SMARCA4 ChIP-seq)

Supp. Figure 7. TAK-981 has activity in synovial sarcoma by inducing DNA damage. A) and **B)** Gene set enrichment analysis (using KEGG (Kyoto Encyclopedia of Genes and Genomes)) of downregulated and upregulated genes detected by ATAC-seq, after treatment of SYO-1 and HS-SY-II cells with TAK-981 for 36h. The *p*-values were adjusted using a False Discovery Rate (FDR) multiple testing correction method. **C)** Whole cell lysates from HS-SY-II, SYO-1 and SS.PDX synovial sarcoma cell lines, treated with 100 nM TAK-981 for 36h were prepared, subjected to immunoblotting, and probed for p-H2AX (Ser 139) and GAPDH. Fig. 1H and Supp. Fig. 7C share the same GAPDH blot (as loading control). n/a: Protein band intensities couldn't be quantified due to the extremely low density of p-H2AX in the untreated (No Rx) lysates. **D)**, **E)** and **F)** Top 20 motifs enriched in the promoters of genes associated with downregulated peaks following TAK-981 treatment from the previous SS18::SSX ChIP-seq data in SYO-1 (Supp. Fig. 7D, the names of the genes written in dark orange), in HS-SY-II (Supp. Fig. 7E, the names of the genes written in neon blue), as well as from the previous SMARCA4 ChIP-seq data in HS-SY-II cells (Supp. Fig. 7F, the names of the genes written in blue).

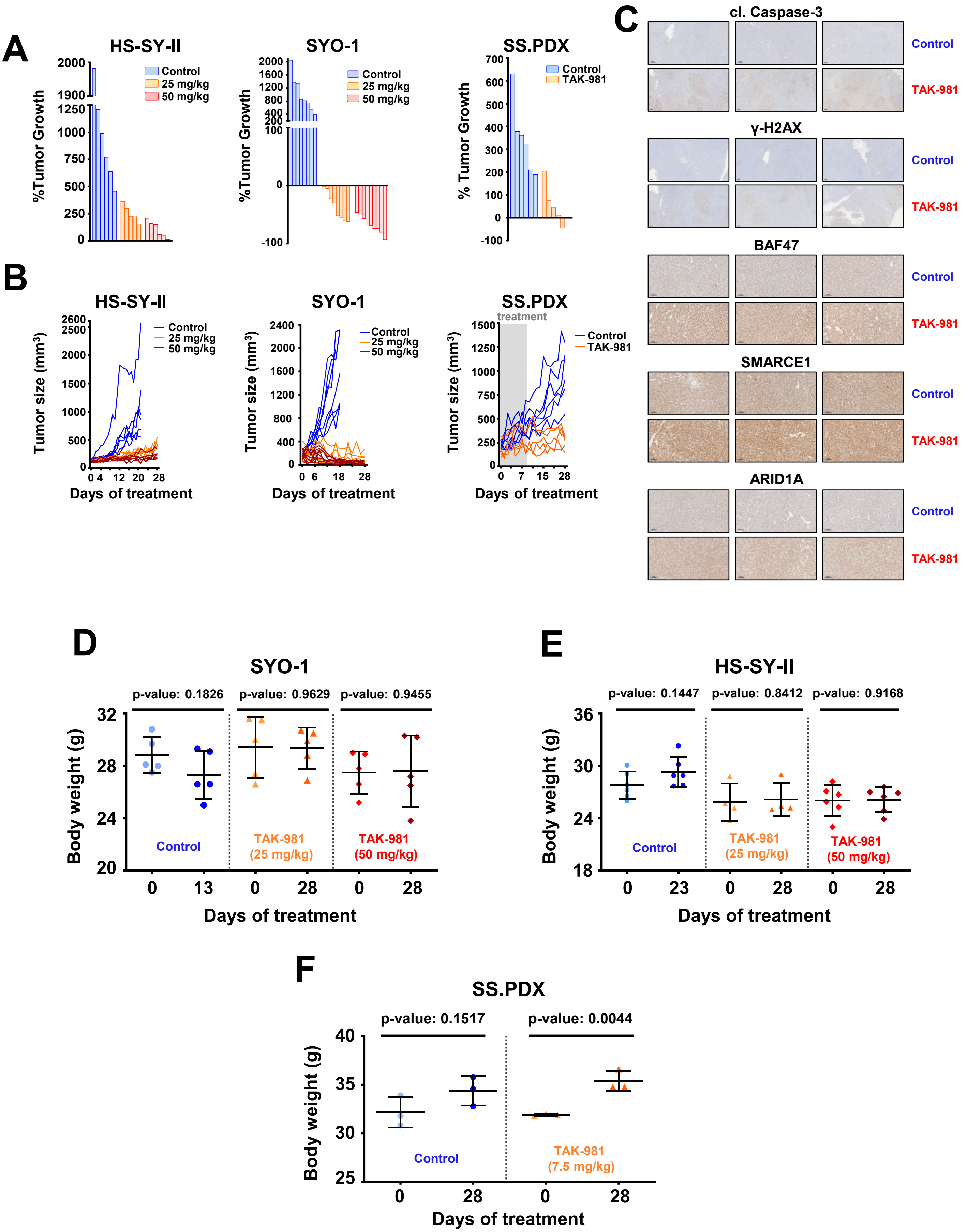
A
HS-SY-II
SYO-1
SS.PDX
%Tumor Growth
% Tumor Growth
Control
25 mg/kg
50 mg/kg
TAK-981
B
Tumor size (mm³)
Days of treatment
treatment
C
cl. Caspase-3
γ-H2AX
BAF47
SMARCE1
ARID1A
Control
TAK-981
D
SYO-1
p-value: 0.1826
p-value: 0.9629
p-value: 0.9455
Body weight (g)
Control
TAK-981
(25 mg/kg)
TAK-981
(50 mg/kg)
Days of treatment
E
HS-SY-II
p-value: 0.1447
p-value: 0.8412
p-value: 0.9168
F
SS.PDX
p-value: 0.1517
p-value: 0.0044
TAK-981
(7.5 mg/kg)

Supp. Figure 8. TAK-981 is effective in different SS mouse models. A) Waterfall plots of HS-SY-II, SYO-1 or SS.PDX tumor-bearing NSG mice (from Fig. 8A, 8B and 8C) were treated with 25mg/kg, 50mg/kg TAK-981 (SYO-1 and HS-SY-II models) or 7.5 mg/kg (SS.PDX model) 3/w or control (no treatment) and tumors were monitored by caliper at least 3/w. **B)** Individual tumors were plotted from Fig. 8A, 8B and 8C. **C)** Representative images of IHC analysis of the indicated antibodies in SYO-1 tumors from the experiment shown in Fig. 8B. Scale bars = 100 µm. **D), E), F)** Weight changes from each of the three experiments from Fig. 8A, 8B, 8C. n=5 (Control), n=5 (25 mg/kg), n=5 (50 mg/kg) for S8D; n=6 (Control), n=4 (25 mg/kg), n=6 (50 mg/kg) for S8E; n=3 (Control), n=3 (TAK-981) for Supp. Fig. 8F (n: number of mice). Unpaired two-sided t tests were performed, and the p-values are shown.

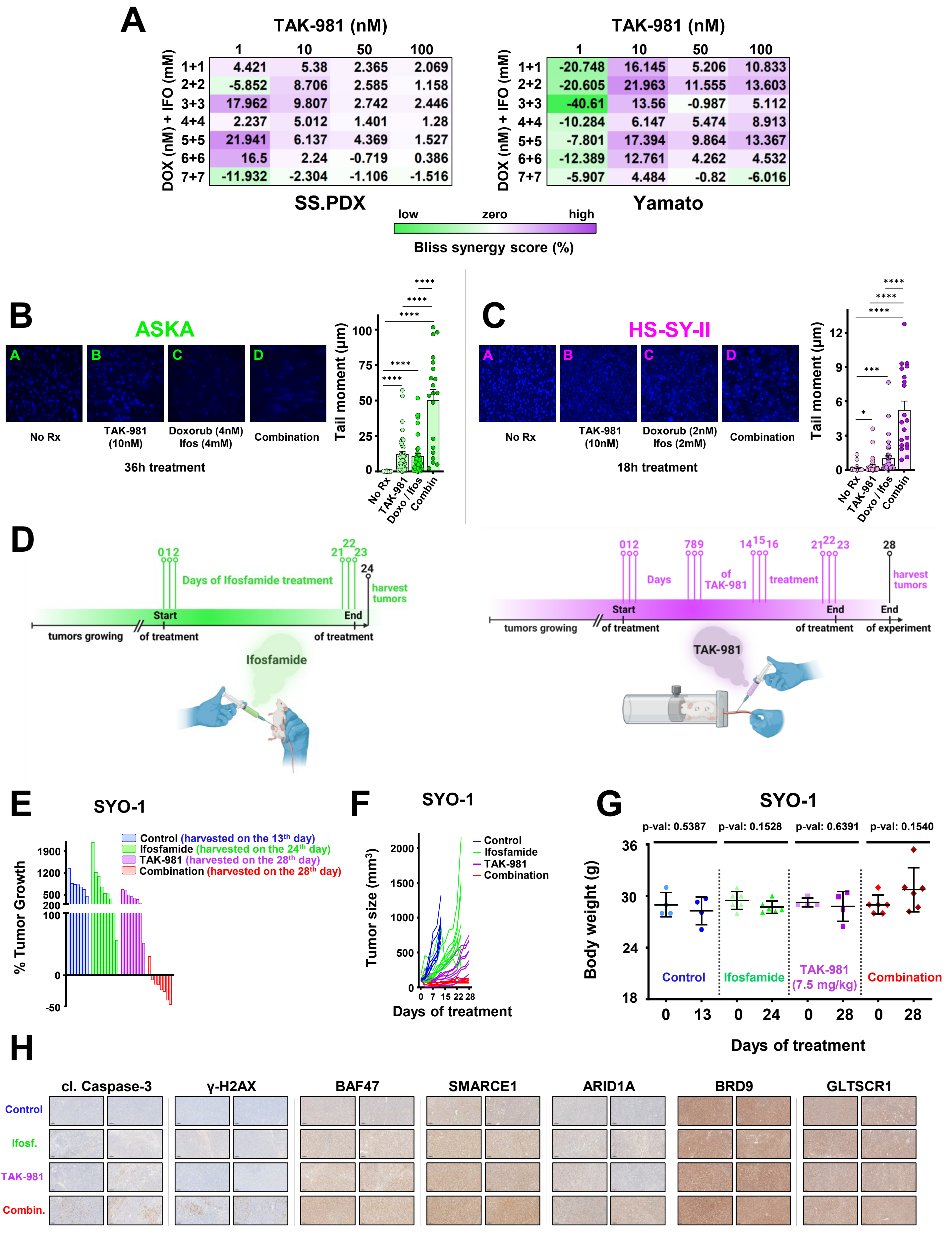
A
TAK-981 (nM)
1 10 50 100
DOX (nM) + IFO (mM)
1+1 4.421 5.38 2.365 2.069
2+2 -5.852 8.706 2.585 1.158
3+3 17.962 9.807 2.742 2.446
4+4 2.237 5.012 1.401 1.28
5+5 21.941 6.137 4.369 1.527
6+6 16.5 2.24 -0.719 0.386
7+7 -11.932 -2.304 -1.106 -1.516
SS.PDX
TAK-981 (nM)
1 10 50 100
DOX (nM) + IFO (mM)
1+1 -20.748 16.145 5.206 10.833
2+2 -20.605 21.963 11.555 13.603
3+3 -40.61 13.56 -0.987 5.112
4+4 -10.284 6.147 5.474 8.913
5+5 -7.801 17.394 9.864 13.367
6+6 -12.389 12.761 4.262 4.532
7+7 -5.907 4.484 -0.82 -6.016
Yamato
low zero high
Bliss synergy score (%)
B
ASKA
A B C D
No Rx
TAK-981 (10nM)
Doxorub (4nM) Ifos (4mM)
Combination
36h treatment
Tail moment (μm)
No Rx TAK-981 Doxo / Ifos Combin
C
HS-SY-II
A B C D
No Rx
TAK-981 (10nM)
Doxorub (2nM) Ifos (2mM)
Combination
18h treatment
Tail moment (μm)
No Rx TAK-981 Doxo / Ifos Combin
D
0 1 2
21 22 23
24
Days of Ifosfamide treatment
harvest tumors
Start
End
tumors growing
of treatment
of treatment
Ifosfamide
0 1 2 7 8 9 14 15 16 21 22 23 28
Days of TAK-981 treatment
harvest tumors
Start
End
End
tumors growing
of treatment
of treatment
of experiment
TAK-981
E
SYO-1
Control (harvested on the 13th day)
Ifosfamide (harvested on the 24th day)
TAK-981 (harvested on the 28th day)
Combination (harvested on the 28th day)
% Tumor Growth
F
SYO-1
Control
Ifosfamide
TAK-981
Combination
Tumor size (mm³)
Days of treatment
G
SYO-1
p-val: 0.5387
p-val: 0.1528
p-val: 0.6391
p-val: 0.1540
Body weight (g)
Control
Ifosfamide
TAK-981 (7.5 mg/kg)
Combination
Days of treatment
H
cl. Caspase-3
γ-H2AX
BAF47
SMARCE1
ARID1A
BRD9
GLTSCR1
Control
Ifosf.
TAK-981
Combin.

Supp. Figure 9. TAK-981 sensitizes SS to cytotoxic chemotherapy *in vitro* and *in vivo* by inducing DNA damage. A) Bliss Sum synergy scores were obtained following treatment for 72hrs with the indicated drugs at the indicated concentrations (like in Fig. 8F). **B)** and **C)** Comet assay was performed following 36h and 18h of the indicated drug treatments (for ASKA and HS-SY-II cells) (combination treatment corresponds to treatment with all three drugs). *n*=40 (No Rx), *n*=40 (TAK-981), *n*=40 (Doxorubicin and Ifosfamide) and *n*=20 (Combination) (*n*: number of cells). Student's *t* tests were performed for all comparisons. **D)** Schema of ifosfamide/TAK-981 treatments from Fig. 8G (Created in BioRender. Floros, K. (2025)). **E)** Waterfall plot of SYO-1 tumor-bearing NSG mice from Fig. 8G. **F)** Individual tumor growth of tumor-bearing NSG mice from Fig. 8G. **G)** Weight changes from the *in vivo* experiment (Fig. 8G). *n*=4 (Control), *n*=5 (Ifosfamide), *n*=4 (TAK-981), *n*=5 (Combination) (*n*: number of mice). Unpaired two-sided *t* tests were performed, and the *p*-values are shown. **H)** Representative images of IHC analysis of the indicated antibodies in SYO-1 tumors from the experiment shown in Fig. 8G, 8H and scored in Fig. 8I.

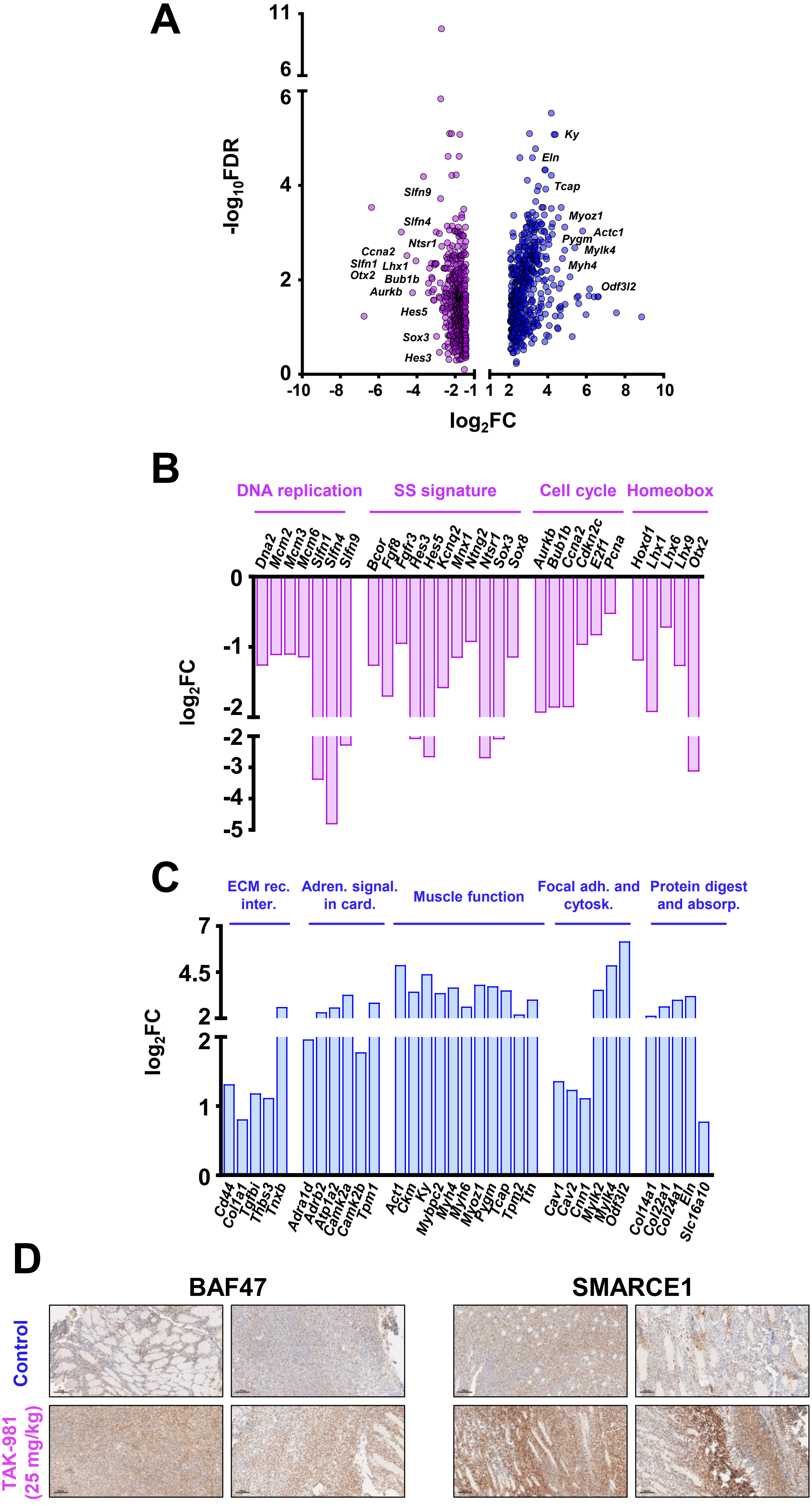
A
-log10FDR
log2FC
Slfn9
Slfn4
Ntsr1
Ccna2
Slfn1
Lhx1
Otx2
Bub1b
Aurkb
Hes5
Sox3
Hes3
Ky
Eln
Tcap
Myoz1
Actc1
Pygm
Mylk4
Myh4
Odf3l2
B
DNA replication
SS signature
Cell cycle
Homeobox
log2FC
Dna2 Mcm2 Mcm3 Mcm6 Slfn1 Slfn4 Slfn9
Bcor Fgf8 Fgfr3 Hes3 Hes5 Kcnq2 Mnx1 Ntng2 Ntsr1 Sox3 Sox8
Aurkb Bub1b Ccna2 Cdkn2c E2f1 Pcna
Hoxd1 Lhx1 Lhx6 Lhx9 Otx2
C
ECM rec. inter.
Adren. signal. in card.
Muscle function
Focal adh. and cytosk.
Protein digest and absorp.
log2FC
Cd44 Col1a1 Tgfbi Thbs3 Tnxb
Adra1d Adrb2 Atp1a2 Camk2a Camk2b Tpm1
Act1 Ckm Ky Mybpc2 Myh4 Myh6 Myoz1 Pygm Tcap Tpm2 Ttn
Cav1 Cav2 Cnn1 Mylk2 Mylk4 Odf3l2
Col14a1 Col22a1 Col24a1 Eln Slc16a10
D
BAF47
SMARCE1
Control
TAK-981 (25 mg/kg)

Supp. Figure 10. SUMOylation pathway inhibition in a conditionally expressed SS18::SSX2 mouse model induces transcriptome changes that are also demonstrated in the *in vitro* data. A) Volcano plot of the 500 most up- and the 500 most down-regulated genes after treatment with TAK-981 in the hSS2 *in vivo* model (from Fig. 9). Some of the most characteristic up- or down-regulated genes are labeled. **B)** Plotted RNA-Seq fold changes of genes downregulated after TAK-981 treatment (Fig. 9E-9G) and their associated pathways related to DNA repair, cell cycle and homeobox TFs. **C)** Plotted RNA-Seq fold changes of genes upregulated after TAK-981 treatment in the hSS2 mice (Fig. 9E-9G), and their associated pathways related to ECM receptor interaction, adrenergic signaling in cardiomyocytes, focal adhesion and protein digestion and absorption. **D)** Representative images of the IHC analysis of the indicated antibodies in the mice from Fig. 9. Scale bars = 100 μm.

1 Beauclair, G., Bridier-Nahmias, A., Zagury, J. F., Saïb, A. & Zamborlini, A. JASSA: a comprehensive tool for prediction of SUMOylation sites and SIMs. *Bioinformatics* **31**, 3483-3491 (2015). https://doi.org/10.1093/bioinformatics/btv403

2 Banito, A. *et al.* The SS18-SSX Oncoprotein Hijacks KDM2B-PRC1.1 to Drive Synovial Sarcoma. *Cancer Cell* **33**, 527-541.e528 (2018). https://doi.org/10.1016/j.ccell.2018.01.018
