# Supplementary material for: Targeting SUMOylation promotes cBAF complex stabilization and disruption of the SS18::SSX transcriptome in synovial sarcoma
Source: Nat Commun. 2025 Nov 5;16:9761. doi: 10.1038/s41467-025-64665-8 (PMC12589557; doi:10.1038/s41467-025-64665-8)
Supplement: Supplementary file 2 — Description of Additional Supplementary Files [file 41467_2025_64665_MOESM2_ESM.pdf]

**Title: Supplementary Data 1**
Description: Results of the proteome-wide detection assay (PTMscan) of the SUMOylation sites in HS-SY-II SS cells before and after TAK-981 treatment (related to Fig. 2A).

**Title: Supplementary Data 2**
Description: Quantification of western densities for Fig. 3D.

**Title: Supplementary Data 3**
Description: Quantification of western densities for Fig. 3E.

**Title: Supplementary Data 4**
Description: qPCR primers for Fig. 2F, 2G, 3C, Supp. Fig. 3J and Supp. Fig. 3L.
