# Supplementary material for: Targeting SUMOylation promotes cBAF complex stabilization and disruption of the SS18::SSX transcriptome in synovial sarcoma
Source: Nat Commun. 2025 Nov 5;16:9761. doi: 10.1038/s41467-025-64665-8 (PMC12589557; doi:10.1038/s41467-025-64665-8)
Supplement: Supplementary file 7 — Reporting Summary [file 41467_2025_64665_MOESM7_ESM.pdf]

nature portfolio

Corresponding author(s): Anthony C. Faber

Last updated by author(s): 8/12/2025

Reporting Summary

Nature Portfolio wishes to improve the reproducibility of the work that we publish. This form provides structure for consistency and transparency in reporting. For further information on Nature Portfolio policies, see our Editorial Policies and the Editorial Policy Checklist.

Statistics

For all statistical analyses, confirm that the following items are present in the figure legend, table legend, main text, or Methods section.

n/a	Confirmed	
☐	☒	The exact sample size (*n*) for each experimental group/condition, given as a discrete number and unit of measurement
☐	☒	A statement on whether measurements were taken from distinct samples or whether the same sample was measured repeatedly
☐	☒	The statistical test(s) used AND whether they are one- or two-sided<br>*Only common tests should be described solely by name; describe more complex techniques in the Methods section.*
☒	☐	A description of all covariates tested
☐	☒	A description of any assumptions or corrections, such as tests of normality and adjustment for multiple comparisons
☐	☒	A full description of the statistical parameters including central tendency (e.g. means) or other basic estimates (e.g. regression coefficient) AND variation (e.g. standard deviation) or associated estimates of uncertainty (e.g. confidence intervals)
☐	☒	For null hypothesis testing, the test statistic (e.g. *F*, *t*, *r*) with confidence intervals, effect sizes, degrees of freedom and *P* value noted<br>*Give P values as exact values whenever suitable.*
☒	☐	For Bayesian analysis, information on the choice of priors and Markov chain Monte Carlo settings
☒	☐	For hierarchical and complex designs, identification of the appropriate level for tests and full reporting of outcomes
☒	☐	Estimates of effect sizes (e.g. Cohen's *d*, Pearson's *r*), indicating how they were calculated

Our web collection on statistics for biologists contains articles on many of the points above.

Software and code

Policy information about availability of computer code

Data collection	Genomics data collection is described in the Data Analysis section.
Data analysis	ChIP-seq<br>Approximately 0.lng to 20ng ChIP DNA was fragmented to size range around 200bps using Covaris (shearing parameter 2 minutes in LV tubes using 15ul microTUBE-15 AFA Beads Screw-cap, Cat: #520145), and used for ChIP-seq library preparation by following the NEBNext Ultra II DNA library preparation Kit for lllumina (Cat. No: E7645L, New England Biolabs Inc, Ipswich, MA, USA). This kit utilizes a proprietary adapter attachment chemistry which minimizes bias and supports very low amount of input sample. ChIP-seq libraries were subjected to quantification process by the combination of Qubit and Bioanalyzer, pooled and subsequent sequenced with either lllumina NovaSeq 6000 or NextSeq 2000 platform. After the sequencing run, demultiplexing with Bcl2fastq2 was employed to generate the fastq file for each sample. The SYO-1 and HS-SY-II SS18-SSX and KDM2B (JHDM1B) ChIP-seq data were quality controlled at each processing step using FASTQC, alignment was performed using STAR v.2.7.9a2 using the UCSC's hg38 genome assembly. Peaks were called with MACS23 and differential peaks were identified using DESeq2 v.1.42.14 using FDR correction for multiple testing. The HS-SY-II SMARCA4 and H3K27ac ChIP-seq data was analysed using the Nextflow ChIP-seq (https://nf-co.re/chipseq/2.0.0) pipeline. Downstream analyses included (differential) motif detections, peak annotation (Chi PPeakAnno v.3.36.16), and functional enrichment analysis of the

associated genes (clusterProfiler v.4.10.17).

RNA-seq
Total RNA was isolated from SYO-1, HS-SY-II and SS.PDX cells using the Isolate IIRNA Mini kit #B1O-52072, Bioline). The quality of Total RNAs was checked by Agilent Tape Station (Agilent Technologies, Santa Clara, CA), and only RNAs with RQN >7 were used for subsequent mRNA-seq library preparation and sequencing. Stranded mRNA-seq library preparation and next generation sequencing: Approximately S00ng Total RNA was used for stranded mRNA-seq library preparation by following the NEB Directional mRNA-seq sample preparation guide (New England
Biolabs, Ipswich, MA). The first step in the workflow involved purifying the poly-A containing mRNA molecules using poly-T oligo-attached magnetic beads. Following purification, the mRNA was fragmented into small pieces using divalent cautions under elevated temperature. The
cleaved RNA fragments were copied into first strand cDNA using reverse transcriptase and random primers. This was followed by second strand cDNA synthesis using DNA Polymerase I and RNase H. Strand specificity was achieved by replacing dTTP with dUTP in the Second Strand Marking Mix (SMM). These cDNA fragments then went through an end repair process, the addition of a single 'A' base, and then ligation of the adapters. The products were then purified and enriched with PCR to create the final RNA-seq library. After RNA-seq libraries were subjected to quantification process, pooled and subsequent 100bp paired read sequencing run with Illumina NovaSeq 6000 platform. After the sequencing run, demultiplexing with Bcl2fastq2 was employed to generate the fastq file, with the average of 30M reads per sample.
The SYO.1 data were aligned to the GRCh38 human genome assembly using bwa v. 0.7.17-r1188 [PMID: 19451168] and gene expression was quantified using featureCounts v. 2.0.1 [PMID: 24227677]. The HS-SY-II data were processed with the Nextflow RNA-seq pipeline (https://nf- co.re/rnaseq/3.14.0). Gene expression was quantified using the STAR-RSEM strategy. The SSPDX RNA-seq data were analyzed by Novogene with us performing differential gene expression analysis. Differentially expressed genes were detected using edgeR v.4.0.168. We also reanalyzed data from Banito et al.9 with limma10 to detect genes differentially expressed between SSX1 and Ren. P-values were adjusted using a False Discovery Rate (FDR) multiple testing correction method11.

Atac seq
ATAC-seq seq library preparation was done by following Active Motif ATAC-Seq Kit Manual (#53150, Active Motif). ATAC-seq libraries were subjected to quantification process by the combination of Qubit and Bioanalyzer, pooled and subsequent sequenced with 100bp paired read sequencing using Illumina NovaSeq 6000 platform. After the sequencing run, demultiplexing with Bcl2fastq2 was employed to generate the fastq file for each sample.
The SYO-1 ATAC-seq data was quality controlled at each processing step using FASTQC, alignment was performed using STAR v.2.7.9a2 using the UCSC's hg38 genome assembly. Peaks were called with MACS23 and differential peaks were identified using DESeq2 v.1.42.14 using FDR correction for multiple testing.
The HS-SY-II ATAC-seq data was analysed using the Nextflow ATAC-seq (https://nf-co.re/atacseq/2.1.2) pipelines, respectively. Downstream analyses included (differential) motif detection5, peak annotation (ChIPPeakAnno v.3.36.16), and functional enrichment analysis of the
associated genes (clusterProfiler v.4.10.17).

For manuscripts utilizing custom algorithms or software that are central to the research but not yet described in published literature, software must be made available to editors and reviewers. We strongly encourage code deposition in a community repository (e.g. GitHub). See the Nature Portfolio guidelines for submitting code & software for further information.

Data

Policy information about availability of data

All manuscripts must include a data availability statement. This statement should provide the following information, where applicable:

- Accession codes, unique identifiers, or web links for publicly available datasets
- A description of any restrictions on data availability
- For clinical datasets or third party data, please ensure that the statement adheres to our policy

All genomics data generated by this study is publicly available in the GEO (Gene Expression Omnibus) database under accession code: GSE266135 (https://www.ncbi.nlm.nih.gov/geo/query/acc.cgi?acc=GSE266135).
The mass spectrometry proteomics data has been deposited to the ProteomeXchange Consortium via the PRIDE partner repository and is publicly available with the dataset identifier: PXD059936 (https://www.ebi.ac.uk/pride/archive/projects/PXD059936).

Research involving human participants, their data, or biological material

Policy information about studies with human participants or human data. See also policy information about sex, gender (identity/presentation), and sexual orientation and race, ethnicity and racism.

Reporting on sex and gender	n/a
Reporting on race, ethnicity, or other socially relevant groupings	n/a
Population characteristics	n/a
Recruitment	n/a
Ethics oversight	n/a

Note that full information on the approval of the study protocol must also be provided in the manuscript.

Field-specific reporting

Please select the one below that is the best fit for your research. If you are not sure, read the appropriate sections before making your selection.

[x] Life sciences [ ] Behavioural & social sciences [ ] Ecological, evolutionary & environmental sciences

For a reference copy of the document with all sections, see nature.com/documents/nr-reporting-summary-flat.pdf

Life sciences study design

All studies must disclose on these points even when the disclosure is negative.

Sample size	Every experiment was performed with at least three biological replicates (n>3). At least three replicates were used for the statistical comparisons. Three or four biological replicates were also used for all the RNA-seq, ChIP-seq and Atac-sec data, to ensure high reliability and reproducibility. For the in vivo experiments at least five tumors per cohort were enrolled in each study to gain meaningful insights.
Data exclusions	No data were excluded from the analyses.
Replication	Every experiment was performed with at least three biological replicates. The data were highly reproducible.
Randomization	For all experiments, samples were allocated to different groups using random assignment to ensure fairness and minimize bias. For the mouse experiments, when tumors reached the indicated size, the tumor-bearing mice were randomized to a no-treatment control group, and the indicated TAK-981 treatment groups. Mice were randomized according to their tumor sizes (at least five grown tumors per cohort) to ensure that the experimental groups are as comparable as possible at the start of the study.
Blinding	Blinding was not relevant in this study.

Reporting for specific materials, systems and methods

We require information from authors about some types of materials, experimental systems and methods used in many studies. Here, indicate whether each material, system or method listed is relevant to your study. If you are not sure if a list item applies to your research, read the appropriate section before selecting a response.

Materials & experimental systems

n/a	Involved in the study	
[ ]	[x]	Antibodies
[ ]	[x]	Eukaryotic cell lines
[x]	[ ]	Palaeontology and archaeology
[ ]	[x]	Animals and other organisms
[x]	[ ]	Clinical data
[x]	[ ]	Dual use research of concern
[x]	[ ]	Plants

Methods

n/a	Involved in the study	
[ ]	[x]	ChIP-seq
[x]	[ ]	Flow cytometry
[x]	[ ]	MRI-based neuroimaging

Antibodies

Antibodies used	The antibodies used for western blotting in this study were as follows: anti-BCL2 (D55G8) (Human Specific) (4223; Cell Signaling), anti-cleaved PARP (Asp214) (D64E10) (5625; Cell Signaling), anti-SMARCE1 (E6H5J) (33360; Cell Signaling), anti-GAPDH (6C5) (sc-32233; Santa Cruz), anti-SMARCB1/BAF47 (D8M1X) (91735; Cell Signaling), anti-BRD9 (24785-1-AP; Proteintech), anti-ARID1A (C-7) (sc-373784; Santa Cruz), anti-PBRM1 (D3F7O) (91894; Cell Signaling), anti-TBP (8515; Cell Signaling), anti-SUMO-2/3 (18H8) (4971; Cell Signaling). For the SUMO detection co-IP, SUMO-2/3 affinity beads (ASM24-Beads; Cytoskeleton) and mouse IgG IP Control Beads (CIG01-Beads; Cytoskeleton) were used. For the SMARCE1 co-IP, anti-BAF57/SMARCE1 antibody (EPR8849) (ab137081; Abcam) and normal rabbit IgG (2729S; Cell Signaling) were used. The secondary antibodies that were used in this study were as follows: ECL Anti-mouse IgG Horseradish Peroxidase conjugated antibody (NA931V; Amersham) and ECL Anti-rabbit IgG Horseradish Peroxidase conjugated antibody (NA934V; Amersham). All primary antibodies were used at 1:1000 dilutions. Secondary antibodies were used at 1:10,000 dilutions.<br>For ChIP seq the following antibodies were used: anti- JHDM1B (KDM2B) (17-10264; Milipore-Sigma), anti-BRG1 (SMARCA4) (EPNCIR111A) (ab110641; Abcam), anti-H3K27ac antibody (pAb) (39134; Active Motif) and anti-SS18::SSX (E9X9V) XP (72364; Cell Signaling) used for both western blotting and ChIP seq.<br>For the IHC staining the following antibodies were used: Cleaved Caspase-3 (1:500 dilution; Cleaved Caspase-3 (Asp175) (5A1)) (Cell Signaling; 9664), ARID1A (1:500 dilution; GeneTex; GTX129433), SMARCE1 (:1000 dilution; Recombinant Anti-BAF57/SMARCE1 antibody [EPR8848]) (Abcam; ab131328), SMARCB1/BAF47 (D8M1X) (1:1500 dilution; Cell Signaling; 91735), p-H2AX (1:400 dilution; Phospho-Histone H2A.X (Ser139) (20E3)) (Cell Signaling; 9718), BRD9 (1:500 dilution; ab259839; Abcam) and GLTSCR1 (1:500 dilution;<br>ab302712; Abcam).
Validation	Validation of the antibodies used in this study has been performed after testing by the commercial vendor, as well as routinely in our laboratory (using genetically KO cell lines).

Eukaryotic cell lines

Policy information about cell lines and Sex and Gender in Research

Cell line source(s)	ASKA (male; Cat # RCB3576), HS-SY-II (male; Cat # RCB2231), and Yamato (male; Cat # RCB3577) cell lines were obtained from Riken Bioresource Center (Tsukuba, Japan). SYO-1 (female) and Fuji (female) were provided by T. Nielsen.
Authentication	Cells were authenticated by quantitative PCR detection of SS18::SSX1/2, which is specific to synovial sarcoma.
Mycoplasma contamination	Routine mycoplasma testing was performed on all cell lines, using the MycoAlert Mycoplasma Detection Kit (LT07–318; Lonza).
Commonly misidentified lines<br>(See ICLAC register)	No commonly misidentified cell lines were used in this study.

Animals and other research organisms

Policy information about studies involving animals; ARRIVE guidelines recommended for reporting animal research, and Sex and Gender in Research

Laboratory animals	6–8-week-old NOD/SCID gamma (NSG) male mice were used for the experiments in Figures 7, S8 and S9<br>(C-G). For the experiment of the hSS2 conditional mouse model in Figures 8 and S10, the mouse strain was maintained on a mixed C57BL/6 and SvJ background with littermate controls and roughly equivalent distribution of sexes. TATCre protein injection was performed in 8 weeks old mice.
Wild animals	No wild animals were used in this study.
Reporting on sex	The findings of the in vivo experiments do not apply to only one sex. Sex was not considered in the study design.
Field-collected samples	This study did not involve samples collected from the field.
Ethics oversight	All mouse experiments of Figures 8, S8 and S9 (D-H) were approved and performed in accordance with the Institutional Animal Care and Use Committee at VCU (protocol number: AD10001048). The hSS2 conditional mouse experiment (Figure 9<br>and S10) was approved by the University of Utah animal care committee in accordance with international legal and ethical standards (protocol number: 00001442).

Note that full information on the approval of the study protocol must also be provided in the manuscript.

Plants

Seed stocks

n/a

Novel plant genotypes

n/a

Authentication

n/a

ChIP-seq

Data deposition

- [x] Confirm that both raw and final processed data have been deposited in a public database such as GEO.
- [x] Confirm that you have deposited or provided access to graph files (e.g. BED files) for the called peaks.

Data access links
May remain private before publication.

All genomics data generated by this study is publicly available in the GEO (Gene Expression Omnibus) database under accession code: GSE266135 (https://www.ncbi.nlm.nih.gov/geo/query/acc.cgi?acc=GSE266135).

Files in database submission

GSE266060 Targeting of SUMOylation leads to cBAF complex stabilization and disruption of the
SS18::SSX transcriptome in
synovial sarcoma [BR_ChIP-seq]
GSE266063 Targeting of SUMOylation leads to cBAF complex stabilization and disruption of the
SS18::SSX transcriptome in synovial sarcoma [HS18_ChIP-seq]
GSE266064 Targeting of SUMOylation leads to cBAF complex stabilization and disruption of the
SS18::SSX transcriptome in synovial sarcoma [HSJHD_ChIP-seq]
GSE266065 Targeting of SUMOylation leads to cBAF complex stabilization and disruption of the
SS18::SSX transcriptome in synovial sarcoma [SY18_ChIP-seq]
GSE266066 Targeting of SUMOylation leads to cBAF complex stabilization and disruption of the
SS18::SSX transcriptome in synovial sarcoma [SYJHD_ChIP-seq]
GSE266134 Targeting of SUMOylation leads to cBAF complex stabilization and disruption of the
SS18::SSX transcriptome in synovial sarcoma [SYATAC_ATAC-seq]
GSE266199 Targeting of SUMOylation leads to cBAF complex stabilization and disruption of the
SS18::SSX transcriptome in synovial sarcoma [HSH3_ChIP-seq_HS-SY-II]
GSE266201 Targeting of SUMOylation leads to cBAF complex stabilization and disruption of the
SS18::SSX transcriptome in synovial sarcoma [HSH3_ChIP-seq_SYO-1]
GSE266203 Targeting of SUMOylation leads to cBAF complex stabilization and disruption of the
SS18::SSX transcriptome in synovial sarcoma [RNA-seq_HS-SY-II]
GSE266206 Targeting of SUMOylation leads to cBAF complex stabilization and disruption of the
SS18::SSX transcriptome in synovial sarcoma [RNA-seq_SYO.1]
GSE266207 Targeting of SUMOylation leads to cBAF complex stabilization and disruption of the
SS18::SSX transcriptome in synovial sarcoma [SSPDX.RNA-seq]
GSE266276 Targeting of SUMOylation leads to cBAF complex stabilization and disruption of the
SS18::SSX transcriptome in synovial sarcoma [RNA-seq_Utah]

Genome browser session
(e.g. UCSC)

n/a

Methodology

Replicates

All ChIP-seq, ATAC-seq, RNA-seq experiments were performed in at least triplicates.

Sequencing depth

Sample_name Raw_read_count Mapped_read_count Uniquely_mapped Length Paired_or_single-
endSY18in.Aligned.sortedByCoord.out 17527749 17527749 14511543 62 Paired-
endSY18tr1.Aligned.sortedByCoord.out 24148299 24148299 20509778 74 Paired-
endSY18tr2.Aligned.sortedByCoord.out 26340187 26340187 22224859 73 Paired-
endSY18tr3.Aligned.sortedByCoord.out 15217025 15217025 12518438 74 Paired-
endSY18un1.Aligned.sortedByCoord.out 10540997 10540997 8321105 74 Paired-

endSY18un2.Aligned.sortedByCoord.out 20545778 20545778 16771964 74 Paired-
endSY18un3.Aligned.sortedByCoord.out 7811028 7811028 6299813 75
Paired-endHS18tr3.Aligned.sortedByCoord.out
16013778 16013778 13089040 76 Paired-endHS18tr1.Aligned.sortedByCoord.out 13235785 13235785
10478280 74 Paired-endHS18tr2.Aligned.sortedByCoord.out 10772602 10772602 8317766 76 Paired-
endHS18in.Aligned.sortedByCoord.out 30437929 30437929 24972828 75 Paired-
endHS18un1.Aligned.sortedByCoord.out 20669817 20669817 16840704 67 Paired-
endHS18un2.Aligned.sortedByCoord.out 24157439 24157439 19544639 75 Paired-
endHS18un3.Aligned.sortedByCoord.out 11195587 11195587 9101600 52 Paired-
endSYATACtr1_S11.Aligned.sortedByCoord.out 22551217 22551217 18012019 75 Paired-
endSYATACtr2_S12.Aligned.sortedByCoord.out 32679509 32679509 26284983 75 Paired-
endSYATACtr3_S13.Aligned.sortedByCoord.out 28755316 28755316 23291663 75 Paired-
endSYATACun1_S8.Aligned.sortedByCoord.out 18090840 18090840 14453967 75 Paired-
endSYATACun2_S9.Aligned.sortedByCoord.out 32231058 32231058 25674440 75 Paired-
endSYATACun3_S10.Aligned.sortedByCoord.out 27477926 27477926 21913350 75 Paired-
endBRtr_REP2.mLb.clN.sorted 61770068 61770068 60361188 60 Paired-endBrun_REP4.mLb.clN.sorted
49667580 49667580 48660614 60 Paired-endBRin.mLb.clN.sorted 43400964 43400964 42434959 60
Paired-endBrun_REP1.mLb.clN.sorted 39531668 39531668 38755488 60
Paired-endBRtr_REP4.mLb.clN.sorted
54432518 54432518 53305047 60 Paired-endBrun_REP2.mLb.clN.sorted 39359396 39359396 38564966
60 Paired-endBrun_REP5.mLb.clN.sorted 40470102 40470102 39671174 60 Paired-
endBRtr_REP3.mLb.clN.sorted 70660202 70660202 69073654 55 Paired-endBRtr_REP5.mLb.clN.sorted
41660566 41660566 40744982 60 Paired-endBrun_REP3.mLb.clN.sorted 40981716 40981716 40176708
59 Paired-endBRtr_REP1.mLb.clN.sorted 36918192 36918192 36126149 60 Paired-
endHSJHDin.Aligned.sortedByCoord.out 18711032 18711032 15646684 73 Paired-
endHSJHDtr1.Aligned.sortedByCoord.out 15350287 15350287 12974996 65 Paired-
endHSJHDtr2.Aligned.sortedByCoord.out 17322564 17322564 14551806 63 Paired-
endHSJHDtr3.Aligned.sortedByCoord.out 15026036 15026036 12666650 53 Paired-
endHSJHDun1.Aligned.sortedByCoord.out 18272245 18272245 15331706 76 Paired-
endHSJHDun2.Aligned.sortedByCoord.out 16280712 16280712 13529506 75 Paired-
endHSJHDun3.Aligned.sortedByCoord.out 17440622 17440622 14697021 36 Paired-
endSYJHDin.Aligned.sortedByCoord.out 20159931 20159931 16558061 67 Paired-
endSYJHDtr1.Aligned.sortedByCoord.out 18246365 18246365 15196304 72 Paired-
endSYJHDtr2.Aligned.sortedByCoord.out 15398792 15398792 12420621 76 Paired-
endSYJHDtr3.Aligned.sortedByCoord.out 18251216 18251216 15046987 74 Paired-
endSYJHDun1.Aligned.sortedByCoord.out 15779210 15779210 12782525 74 Paired-
endSYJHDun2.Aligned.sortedByCoord.out 18497303 18497303 15317373 65 Paired-
endSYJHDun3.Aligned.sortedByCoord.out 18045617 18045617 14649403 64
Paired-endHSH3in.mLb.clN.sorted
44861738 44861738 43894128 59 Paired-endHSH3tr_REP1.mLb.clN.sorted 41084490 41084490
40509692
59 Paired-endHSH3tr_REP2.mLb.clN.sorted 39681496 39681496 39078600 60 Paired-
endHSH3tr_REP3.mLb.clN.sorted 36075306 36075306 35532834 60
Paired-endHSH3tr_REP4.mLb.clN.sorted
53067146 53067146 52228160 60 Paired-endHSH3un_REP1.mLb.clN.sorted 38171318 38171318
37594746
60 Paired-endHSH3un_REP2.mLb.clN.sorted 34063614 34063614 33558305 60 Paired-
endHSH3un_REP3.mLb.clN.sorted 46258768 46258768 45574797 60
Paired-endHSH3un_REP4.mLb.clN.sorted
20201040 20201040 19857174 60 Paired-endSYH3in_S7.Aligned.sortedByCoord.out 35146041 35146041
29090253 75 Paired-endSYH3tr1_S4.Aligned.sortedByCoord.out 39154480 39154480 34586351 75
Paired-
endSYH3tr2_S5.Aligned.sortedByCoord.out 31881119 31881119 28452399 75 Paired-
endSYH3tr3_S6.Aligned.sortedByCoord.out 33426678 33426678 29421550 75 Paired-
endSYH3un1_S1.Aligned.sortedByCoord.out 33273864 33273864 28931045 75 Paired-
endSYH3un2_S2.Aligned.sortedByCoord.out 51136000 51136000 45567652 75 Paired-
endSYH3un3_S3.Aligned.sortedByCoord.out 36338443 36338443 32391767 75
Paired-endHSRNAtr1.markdup.sorted
79388798 77767236 60054857 59 Paired-endHSRNAtr2.markdup.sorted 83615023 81932270 63002698
60 Paired-endHSRNAtr3.markdup.sorted 73065180 71567051 55319996 60
Paired-endHSRNAtr4.markdup.sorted
84236763 82483993 63587447 60 Paired-endHSRNAun1.markdup.sorted 83362895 82365537 66129572
60 Paired-endHSRNAun2.markdup.sorted 80212314 79220059 63278434 60
Paired-endHSRNAun3.markdup.sorted
72730929 71662712 56618029 60 Paired-endHSRNAun4.markdup.sorted 69379001 68468350 54070373
59 Paired-endTr48h_1_bwa 69637969 69623066 63224053 150 Paired-endTr48h_2_bwa 69565259
69550288 63460774 55 Paired-endTr48h_3_bwa 55274708 55263718 50131937 150 Paired-
endTr72h_1_bwa 56703846 56694143 51759636 150 Paired-endTr72h_2_bwa 73039995 73024376
66432140 150 Paired-endTr72h_3_bwa 78122836 78110392 71354897 150 Paired-endUntreated_1_bwa

72379202 72368274 65940932 57 Paired-endUntreated_2_bwa 67460136 67449275 61305855 150
Paired-endUntreated_3_bwa 72309870 72295575 65667072 30 Paired-end

Antibodies

For ChIP seq the following antibodies were used: anti- JHDM1B (KDM2B) (17-10264; Milipore-Sigma), anti-BRG1 (SMARCA4) (EPNCIR111A) (ab110641; Abcam), anti-H3K27ac antibody (pAb) (39134; Active Motif) and anti-SS18::SSX (E9X9V) XP (72364; Cell Signaling) used for both western blotting and ChIP seq.

Peak calling parameters

The SYO-1 and HS-SY-11 S518-SSX and KDM2B (JHDM1B) ChIP-seq data were quality controlled at each processing step using FASTQC, alignment was performed using STAR v.2.7.9a2 using the UCSC's hg38 genome assembly. Peaks were called with MACS23 and differential peaks were identified using DESeq2 v.1.42.14 using FDR correction for multiple testing. The HS-SY-11 SMARCA4 and H3K27ac ChIP-seq data was analysed using the Nextflow ChIP-seq (https://nf-co.re/chipseq/2.0.0) pipeline.

The SYO-1 ATAC-seq data was quality controlled at each processing step using FASTQC, alignment was performed using STAR
v.2.7.9a2 using the UCSC's hg38 genome assembly. Peaks were called with MACS23 and differential peaks were identified using DESeq2 v.1.42.14 using FDR correction for multiple testing.
The HS-SY-11 ATAC-seq data was analysed using the Nextflow ATAC-seq (https://nf-co.re/atacseq/2.1.2) pipelines, respectively.

Data quality

Sample Peak_CountSY18tr1_vs_ín_peaks.narrowPeak 50773SY18tr2_vs_ín_peaks.narrowPeak
47412SY18tr3_vs_ín_peaks.narrowPeak 44136SY18un1_vs_ín_peaks.narrowPeak
32296SY18un2_vs_ín_peaks.narrowPeak 45734SY18un3_vs_ín_peaks.narrowPeak
263591HS18ín_vs_ín_peaks.narrowPeak 9414HS18tr1_vs_ín_peaks.narrowPeak
55370HS18tr2_vs_ín_peaks.narrowPeak 29369HS18tr3_vs_ín_peaks.narrowPeak
56424HS18un1_vs_ín_peaks.narrowPeak 104607HS18un2_vs_ín_peaks.narrowPeak
63668HS18un3_vs_ín_peaks.narrowPeak 55219SYATACtrl_S11_peaks.narrowPeak
76655SYATACtr2_S12_peaks.narrowPeak 86880SYATACtr3_S13_peaks.narrowPeak
80439SYATACtr_peaks.narrowPeak 118495SYATACun1_S8_peaks.narrowPeak
45325SYATACun2_S9_peaks.narrowPeak 53108SYATACun3_S10_peaks.narrowPeak
51593SYATACun_peaks.narrowPeak 75624BRtr_REP1_peaks.narrowPeak 7986BRtr_REP2_peaks.narrowPeak
15393BRtr_REP3_peaks.narrowPeak 30191BRtr_REP4_peaks.narrowPeak 37382BRtr_REP5_peaks.narrowPeak
11052Brun_REP1_peaks.narrowPeak 9856Brun_R EP2_peaks.narrowPeak 2281Brun_R EP3_peaks.narrowPeak
4568 Brun_REP4_peaks.narrowPeak 3325Brun_R EP5_peaks.narrowPeak 2929
HSJHDin_vs_ín_peaks.narrowPeak
2957HSJ HDtrl_vs_ín_peaks.narrowPeak 31485HSJ HDtr2_vs_ín_peaks.narrowPeak
13744HSJHDtr3_vs_ín_peaks.narrowPeak 28948HSJ HDun1_vs_ín_peaks.narrowPeak 10804HSJ
HDun2_vs_ín_peaks.narrowPeak 20060HSJHDun3_vs_ín_peaks.narrowPeak 30349SYJ
HDín_vs_ín_peaks.narrowPeak 3914SYJH Dtrl_vs_ín_peaks.narrowPeak 38354SYJ
HDtr2_vs_ín_peaks.narrowPeak 39500SYJ HDtr3_vs_ín_peaks.narrowPeak
40867SYJHDun1_vs_ín_peaks.narrowPeak 36218SYJ HDun2_vs_ín_peaks.narrowPeak 18974SYJ
HDun3_vs_in_peaks.narrowPeak 60288HSH3tr_REPl_peaks.narrowPeak 62150HSH3tr_REP2_peaks.narrowPeak
58605HSH3tr_REP3_peaks.narrowPeak 57256HSH3tr_REP4_peaks.narrowPeak
76143HSH3un_REP1_peaks.narrowPeak 40188HS H 3un_REP2_peaks.narrowPeak 40028HSH3un_RE
P3_peaks.narrowPeak 58826HS H 3un_REP4_peaks.narrowPeak 30896SYH3in_S7_vs_in_peaks.narrowPeak
7167SYH3tr1_S4_vs_in_peaks.narrowPeak 6739lSYH3tr2_S5_vs_ín_peaks.narrowPeak
75570SYH3tr3_S6_vs_in_peaks.narrowPeak 80444SYH3un1_S1_vs_ín_peaks.narrowPeak
56897SYH3un2_S2_vs_ín_peaks.narrowPeak 42546SYH3un3_S3_vs_in_peaks.narrowPeak   48638

Software

ChIP seq
The SYO-1 and HS-SY-11 5S18-SSX and KDM2B (JHDM1B) ChIP-seq data were quality controlled at each processing step using FASTQC, alignment was performed using STAR v.2.7.9a2 using the UCSC's hg38 genome assembly. Peaks were called with MACS23 and differential peaks were identified using DESeq2 v.1.42.14 using FDR correction for multiple testing. The HS-SY-11 SMARCA4 and H3K27ac ChIP-seq data was analysed using the Nextflow ChIP-seq (https://nf-co.re/chipseq/2.0.0) pipeline. Downstream analyses included (differential) motif detection5, peak annotation (ChIPPeakAnno v.3.36.16), and functional enrichment analysis of the associated genes (clusterProfiler v.4.10.17).

RNA seq
The SYO.1 data were aligned to the GRCh38 human genome assembly using bwa v. 0.7.17-r1188 [PMID: 19451168] and gene expression was quantified using featureCounts v. 2.0.1 [PMID: 24227677]. The HS-SY-11 data were processed with the Nextflow RNA- seq pipeline (https://nf-co.re/rnaseq/3.14.0). Gene expression was quantified using the STAR-RSEM strategy. The SSPDX RNA-seq data were analyzed by Novogene with us performing differential gene expression analysis. Differentially expressed genes were detected using edgeR v.4.0.168. We also reanalyzed data from Banito et al.9 with limma10 to detect genes differentially expressed between SSX1 and Ren. P-values were adjusted using a False

Discovery Rate (FDR) multiple testing correction method11.

Atac seq
The SYO-1 ATAC-seq data was quality controlled at each processing step using FASTQC, alignment was performed using STAR
v.2.7.9a2 using the UCSC's hg38 genome assembly. Peaks were called with MACS23 and differential peaks were identified using DESeq2 v.1.42.14 using FDR correction for multiple testing.
The HS-SY-11 ATAC-seq data was analysed using the Nextflow ATAC-seq (https://nf-co.re/atacseq/2.1.2) pipelines, respectively. Downstream analyses included (differential) motif detection5, peak annotation (ChIPPeakAnno v.3.36.16), and functional enrichment
analysis of the associated genes (clusterProfiler v.4.10.17).
